# Dynamic surplus optimization with performance- and index-linked liabilities

**DOI:** 10.1007/s13385-021-00292-z

**Published:** 2021-08-24

**Authors:** Sascha Desmettre, Markus Wahl, Rudi Zagst

**Affiliations:** 1 Johannes Kepler University Linz, Altenberger Straße 69, 4040 Linz, Austria; 2 Technical University of Munich, Parkring 11, 85748 Garching, Germany

**Keywords:** Surplus optimization, Asset-liability management, Performance participation, Stochastic liabilities, Random utility, Martingale method

## Abstract

The increasing importance of liability-driven investment strategies and the shift towards retirement products with lower guarantees and more performance participation provide challenges for the development of portfolio optimization frameworks which cover these aspects. To this end, we establish a general and flexible terminal surplus optimization framework in continuous time, allowing for dynamic investment strategies and stochastic liabilities, which can be linked to the performance of an index or the asset portfolio of the insurance company. Besides optimality results in a fairly general surplus optimization setting, we obtain closed-form solutions for the optimal investment strategy for various specific liability models, which include the cases of index-linked and performance-linked liabilities and liabilities which are completely or only partially hedgeable. We compare the results in numerical examples and study the impact of the performance participation, unhedgeable risk components, different ways of modeling the liabilities and the relative risk aversion parameter. We find that performance- or index-linked liabilities, which provide a close link between the wealth of the insurance company and its liabilities, allow for a higher allocation in the risky investment. On the other hand, unhedgeable risks reduce the allocation in the risky investment. We conclude that, aiming at a high expected return for the policy holder, insurance companies should try to connect the performance of insurance products closely to the wealth and minimize unhedgeable risks.

## Introduction

In recent times, among others, the following two important developments for insurance companies as well as for pension funds, have become more and more evident:

1. An increasing importance of liability-driven investment strategies.
2. A shift towards retirement products which provide less guarantees, but more participation in the performance of certain assets to the policy holder. We consider products which offer a participation in the wealth of the insurance company itself (performance-linked products) and products, which are linked to the performance of an external fund or index (index-linked products).

This paper addresses these demands by establishing a terminal surplus optimization with dynamic investment strategies, in which we also investigate liabilities which depend on the performance of the assets. Applying a generalized martingale approach, analytically tractable solutions for the optimal investment strategy can generally not be found. We obtain closed-form solutions for the optimal investment strategy for specific liability models (including index-linked, performance-linked and partially unhedgeable risks) as key contributions. Consequently, our paper contributes to three literature strands:

Firstly, we extend previous results on portfolio optimization with stochastic liabilities. Starting with [20], the (partial) surplus optimization problem has been addressed in a one-period financial market with a static investment strategy. In continuous time, stochastic liabilities have, e.g., been dealt with by maximizing the utility of the intertemporal surplus [17] or the terminal funding ratio [16]. Josa-Fombellida and Rincón-Zapatero [10] consider defined benefit pension plans and the possibility to manage the rate of contribution in addition to the investment strategy. In more recent times, various different approaches to liability-driven investments were considered and extended in [3]. Closer to our work, [5] directly aim at transforming the surplus optimization from [20] to a setting in continuous time with a maximization of the utility from the excess of liquid wealth over a minimum liability coverage. However, they focus on the inclusion of various stochastic factors and numerical results, which are obtained using simulation, in contrast to our setting which covers partially hedgeable liabilities and allows for closed-form solutions in a number of liability models.

Secondly, we contribute to the literature of insurance products, for which the policy holder participates in gains from the investment strategy of the insurance company, as they are, e.g., described in [14]. Closer to the mentioned literature in the more specific surplus setting [17, 20], the work of [12] investigates how different surplus distribution mechanisms affect the risk exposure of life insurance companies that sell performance-participating life insurance contracts. [15] obtain closed-form solutions for optimal investment strategies with performance-participating liabilities in a complete market for S-shaped utility functions. By optimizing the terminal surplus in the presence of index- and performance-linked liabilities, which might include unhedgeable risks as well, our paper establishes a link between the literature on portfolio optimization with stochastic liabilities and the literature on insurance products.

Finally, we also contribute to the literature on portfolio optimization with random utility functions and with random endowment. Random utility functions have for instance been utilized in order to determine optimal portfolios with a positive lower bound on the terminal wealth in [13] or taking deferred capital gains taxes into account in [19]. The abstract solution to expected utility maximization problems of agents that receive a random endowment at maturity can be found in [8, 9], who use general duality methods. In this paper, instead of those general duality methods, we apply the martingale ansatz to derive optimal solutions for a surplus optimization which includes liabilities in the classical optimization of terminal wealth. Moreover, in contrast to the abstract existence and uniqueness results of [8, 9], we give explicit solutions of optimal investment strategies for different types of liabilities by exploiting specific liability models.

Our work is also closely related to [4], in which the authors develop a two-step method for a setting that includes an illiquid asset which can only be traded at the beginning of the time horizon. In the first step, the authors find an optimal liquid portfolio given an arbitrary, but fixed illiquid investment and in the second step, they optimize the amount invested in the illiquid asset. We extend the methods used in the first step to our setting by interpreting the liability position as a *short* position in an illiquid asset and allowing for such a short position. Furthermore, we derive closed-form solutions for the optimal trading strategies in the setting of replicable liabilities and for various types of liabilities with performance participation.

This paper is structured as follows: In Sect. 2, we extend the (partial) surplus optimization approach from [20] to a framework in continuous time by adapting the optimization approach presented in [4]. The approach is then used to find optimal investment strategies with replicable liabilities in Sect. 3. In Sect. 4, liabilities which depend on the performance of the asset portfolio of the insurance company and which allow for unhedgeable risks are studied. The optimal investment strategies are compared in Sect. 5 and a conclusion is provided in Sect. 6.

## Dynamic surplus optimization approach

In this chapter, we introduce the financial market model and describe the surplus optimization approach.

### Model for assets and liabilities

To model the assets and liabilities, we consider a probability space $({\varOmega },{\mathscr {H}},{\mathbb {Q}})$, with ${\mathscr {H}}={\mathscr {F}}\vee {\mathscr {G}}$ and ${\mathscr {F}}$ and ${\mathscr {G}}$ independent. We further assume that ${\mathbb {F}} {=} ({\mathscr {F}}_{t \in [0,T]})$ is a filtration on ${\mathscr {F}}$, generated by a Brownian motion *W* satisfying the usual conditions and $T>0$ denotes the time horizon for the investor. *W* is used to model the risky asset and hedgeable liability risks and ${\mathscr {G}}$-measurable random variables are used to model the unhedgeable risks. We introduce the risk-free asset $P_0(t)$ satisfying

$$\begin{aligned} dP_0(t) = P_0(t) r(t) dt, \end{aligned}$$

with *r*(*t*) being a non-negative, absolutely-integrable, deterministic function on [0, *T*]. We also assume that the investor can invest in a risky asset *P* following the dynamics

1 $$\begin{aligned} dP(t)=P(t)\left( \mu (t) dt + \sigma (t) dW(t)\right) , \end{aligned}$$

where $\mu (t)$ is an absolutely-integrable, and $\sigma (t)$ is a square-integrable, strictly positive, deterministic function on [0, *T*]. Depending on the application, we may also restrict $\mu (t),\sigma (t)$ and *r*(*t*) to be constant. ${\tilde{Z}}(t)$ denotes the pricing kernel in this complete market given by

$$\begin{aligned} {\tilde{Z}}(t) := e^{-\int _0^t r(s)+\frac{1}{2}\gamma ^2(s)ds - \int _0^t\gamma (s)dW(s)}, \end{aligned}$$

with $\gamma (t) := (\mu (t)-r(t))/\sigma (t)$ being the market price of risk. We assume that the pricing kernel fulfills ${\mathbb {E}}[{\tilde{Z}}(T)] < \infty$ and we write

$$\begin{aligned} {\tilde{Z}}(t,T):=\frac{{\tilde{Z}}(T)}{{\tilde{Z}}(t)}. \end{aligned}$$

We consider ${\mathbb {F}}$-progressively measurable, self-financing investment strategies with $\pi (t)$ being the fraction of wealth invested in the risky asset at time $t\in [0,T]$. The remaining portion $1- \pi (t)$ is invested in the money market account. The wealth process is denoted by *V*(*t*) and the initial wealth by $V(0)=v_0>0$. The dynamics of *V*(*t*) are given by

2 $$\begin{aligned} dV(t) = V(t)\left( (r(t) + \pi (t)(\mu (t)-r(t)))dt + \pi (t)\sigma (t)dW(t)\right) . \end{aligned}$$

We assume

$$\begin{aligned} \int _0^T \pi ^2(s)V^2(s)ds < \infty , \quad {\mathbb {Q}}\text {-a.s.} \end{aligned}$$

To set up the portfolio optimization problem, we first introduce the liabilities in a way which is inspired by the illiquid asset in [4]. We assume that the value of the liabilities at time *T* can be modeled by random variables $L_1(T)$ and $L_2(T)$ and that we cannot invest in the liabilities directly. $L_1(T)$ will be used to model a performance-participating part and $L_2(T)$ will be used to model a part which is not directly depending on the wealth. $L_2(T)$ can be interpreted, e.g., as an index-linked part. For both, $L_1$ and $L_2$, we want to allow for hedgeable and non-hedgeable components. The hedgeable risks can be interpreted as, e.g., interest-rate risks and the unhedgeable risks as, e.g., inflation risks (income growth of the policy holder), mortality risk or operational risk inherent in the liabilities. The hedgeable components are modeled by a stochastic process *X*, which satisfies the following assumption.

#### Assumption (LX)

The process *X* follows

$$\begin{aligned} dX(t)=\mu _X(t,X(t))dt+\sigma _X(t,X(t))dW(t) \end{aligned}$$

and the SDE has a unique solution. Furthermore, we assume that, for $t\in [0,T]$, *X*(*T*) can be written as a function of *X*(*t*) and an increment $\xi (t,T)$, i.e.

$$\begin{aligned} X(T)=g(X(t),\xi (t,T)), \end{aligned}$$

where $\xi (t,T)$ is independent of *X*(*t*) and *g* is twice differentiable with respect to the first component.

Note that *X*(*T*) is replicable since *X* is driven by the same Brownian motion *W* as the risky asset *P* from (1). We assume that the investor cannot trade in *X*, only in the risky asset. Therefore, the model is also free of arbitrage. In some applications, we assume that *X* follows a geometric Brownian motion as stated in the following example.

#### Example 1

(Geometric Brownian Motion) We assume that *X* follows a geometric Brownian motion

3 $$\begin{aligned} dX(t) = X(t)\left( {\hat{\mu }}_X dt + {\hat{\sigma }}_X dW(t)\right) ,\,X(0)=1, \end{aligned}$$

with constant coefficients ${\hat{\mu }}_X\in {\mathbb {R}}$, ${\hat{\sigma }}_X>0$ and *W* from (1). Since

$$\begin{aligned} X(T) =&\, X(t) e^{ \left( {\hat{\mu }}_X - \frac{1}{2} {\hat{\sigma }}_X^2 \right) (T-t) + {\hat{\sigma }}_X \left( W(T) - W(t) \right) }, \end{aligned}$$

$X(T)=g(X(t),\xi (t,T))$ with $\xi (t,T)=e^{ \left( {\hat{\mu }}_X - \frac{1}{2} {\hat{\sigma }}_X^2 \right) (T-t) + {\hat{\sigma }}_X \left( W(T) - W(t) \right) }$ and $g(x,\xi )=x\xi$. Hence, Assumption (LX) is satisfied.

To model the unhedgeable components of the liabilities, we use ${\mathscr {G}}$-measurable random variables ${\mathscr {U}}_1$ and ${\mathscr {U}}_2$. We consider liabilities *L*(*T*, *v*) which fulfill the following Assumptions (L1) and (L2). Assumption (L1) states the general structure of the liabilities. Assumption (L2) contains conditions to exclude the possibility of unavoidable bankruptcy.

#### Assumption (L1)

The liabilities are of the form

L1 $$\begin{aligned} L(T,v)=L(T,v,X(T),{\mathscr {U}}_1,{\mathscr {U}}_2)=vL_1(T,X(T),{\mathscr {U}}_1)+L_2(T,X(T),{\mathscr {U}}_2),\,v>0, \end{aligned}$$

with non-negative, ${\mathscr {H}}$-measurable functions $L_i(T,X(T),{\mathscr {U}}_i)$, $i=1,2$.

We call the left part of the sum $vL_1(T,X(T),{\mathscr {U}}_1)$ *performance-linked* and the right part $L_2(T,X(T),{\mathscr {U}}_2)$ *index-linked*. To simplify the notation, we will write *L*(*T*) or *L*(*T*, *v*) instead of $L(T,v,X(T),{\mathscr {U}}_1,{\mathscr {U}}_2)$ and $L_i(T)$ instead of $L_i(T,X(T),{\mathscr {U}}_i)$, $i=1,2$ sometimes. For each $\omega \in {\varOmega }$, we further assume the existence of the worst case scenario with respect to the unhedgeable risks defined by

$$\begin{aligned} {\hat{\omega }}_i:=\arg \sup _{{\hat{\omega }}\in {\varOmega }}L_i(T,X(T,\omega ),{\mathscr {U}}_i({\hat{\omega }})),\,i=1,2. \end{aligned}$$

We want to maximize the expected utility of the surplus of the assets over the liabilities at time *T* and thus define the surplus as introduced in [20] by

$$\begin{aligned} S(T):=V(T)-\psi _LL(T,V(T)), \end{aligned}$$

where the factor $\psi _L\in (0,1]$ is constant as in [20] and allows us to consider a flexible portion of the liabilities. In *T*, the liabilities are covered even for the worst outcomes of the unhedgeable risk components, if

4 $$\begin{aligned} V(T,\omega )\ge \psi _LV(T,\omega )L_1(T,X(T,\omega ),{\mathscr {U}}_1({\hat{\omega }}_1))+\psi _LL_2(T,X(T,\omega ),{\mathscr {U}}_2(\hat{\omega _2})), \end{aligned}$$

i.e. if $$V(T,\omega )\ge {\hat{v}}_0(\omega )$$ with

5 $$\begin{aligned} {\hat{v}}_0(\omega ):=\frac{\psi _LL_2(T,X(T,\omega ),{\mathscr {U}}_2({\hat{\omega }}_2))}{1-\psi _LL_1(T,X(T,\omega ),{\mathscr {U}}_1({\hat{\omega }}_1))}. \end{aligned}$$

#### Assumption (L2)

There exists a constant $k_1\in \left[ 0,\frac{1}{\psi _L}\right)$ and a random variable $k_2(\omega )$ such that ${\mathbb {E}}\left[ {\tilde{Z}}(T)k_2(\omega )\right] <\infty$ and ${\mathbb {Q}}\text {-a.s.}$

L2.1 $$\begin{aligned} k_1 \ge&L_1(T,X(T),{\mathscr {U}}_1) \end{aligned}$$

L2.2 $$\begin{aligned} \text {and }k_2(\omega )\ge&\sup _{{\hat{\omega }}\in {\varOmega }}L_2(T,X(T,\omega ),{\mathscr {U}}_2({\hat{\omega }})) \end{aligned}$$

hold. Furthermore,

L2.3 $$\begin{aligned} v_0\ge {\mathbb {E}}\left[ {\tilde{Z}}(T){\hat{v}}_0(\omega )\right] . \end{aligned}$$

In particular, this means that the investor has enough initial capital to hedge the worst outcomes of the unhedgeable risk components associated with ${\mathscr {U}}_i$, $i=1,2$. For a pension plan or insurance company with liabilities consisting of the discounted cashflows of the future payments, an upper bound could be, e.g., the sum of all (non-discounted) payments or a cap in benefits to the policy holders (see [6]). For a discussion of optimal investment strategies in a liability-driven investment context with underfunding, which is not covered here, see e.g. [1, 10] or [3]. A self-financing strategy $\pi (t)$ is called admissible if

6 $$\begin{aligned} V(t)-{\mathbb {E}}\left[ {\tilde{Z}}(t,T){\hat{v}}_0(\omega )|{\mathscr {F}}_t\right] \ge 0 \; {\mathbb {Q}}\text{-a.s. } \text{ for } \text{ all }\, t \in [0,T] \end{aligned}$$

and ${\mathbb {E}}\left[ U^-(S(T))\right] <\infty$. We denote the set of all admissible strategies corresponding to initial wealth $v_0$ by ${\varLambda }(v_0)$.

#### Remark 1

For $V(T,\omega )\ge {\hat{v}}_0(\omega )$ ${\mathbb {Q}}$-a.s., the investor has enough capital to cover the worst outcomes of the unhedgebale risks in *T* (see (4) and (5)). Consequently, if (6) holds, the investor has enough capital to cover the present value of the liabilities with respect to the worst outcomes of the unhedgeable risk components in $t\in [0,T]$. Note that we assume that the investor has enough initial capital (see Assumption (L2.3)).

### Specific liability models

In this section, we present some specific liability models, for which we obtain more explicit results later. We also discuss Assumptions (LX), (L1) and (L2), where (L2) is always evaluated assuming that the investor has enough initial capital, i.e. (L2.3) holds. In order to make clear which type of liabilities we are considering at each point, we use the following abbreviations: performance-linked liabilities are indicated by ’PL’, index-linked-liabilities by ’IL’ and liabilities which include both components by ’PIL’. We furthermore use the following additions: ’R’ for replicable liabilities, ’U’ to indicate that there are unhedgeable components and ’CB’ for capped benefits.

#### Example 2

(Replicable liabilities (PILR)) If we do not consider unhedgeable risks associated with ${\mathscr {U}}_i,\,i=1,2$, but a general process *X* satisfying Assumption (LX) as well as general $L_1(T)$ and $L_2(T)$ satisfying Assumption (L2), we have

PILR $$\begin{aligned} L(T,v)=vL_1(T)+L_2(T)=vL_1(T,X(T))+L_2(T,X(T)). \end{aligned}$$

In this case, we can set $k_2(\omega ):=L_2(T,X(T,\omega ))$.

#### Example 3

(Index-linked liabilities with capped maximum benefits (ILRCB)) Motivated by [6], we consider liabilities of the form

ILRCB $$\begin{aligned} L_1(T)=0,\;\; L(T,X(t))=L_2(T,X(T))= L(0) f(X(T)),\,L(0)>0,\,t\in [0,T], \end{aligned}$$

with capped maximum benefits, i.e.

7 $$\begin{aligned} f(x) = \min \left\{ x,K\right\} \end{aligned}$$

and *X* as in Example 1. This type of liabilities satisfies Assumptions (LX), (L1) and (L2) with $L_1(T)=0$, $L_2(T,X(t))=L(0)f(X(T))$, $k_1=0$ and $k_2(\omega )\equiv L(0)K$. The cap can be interpreted, e.g., as a special product feature to limit the insurance company’s risk (see [6]), as an implicit guarantee by the supervising authority to change rules in case of industry-wide underfunding, or as some form of natural upper-bound as described in Sect. 2.1.

In the following examples, we assume that the payment to the policy holder is depending on the performance of the asset portfolio of the insurance company. Such mechanisms can be found in various types of insurance contracts, see e.g. [14] or [12].

#### Example 4

(Performance-linked liabilities (PLU)) We consider performance-linked liabilities with unhedgeable risks of the form

PLU $$\begin{aligned} L_2(T)=0,\;\; L(T,v)=vL_1(T,X(T),{\mathscr {U}}_1), \end{aligned}$$

with general, $L_1(T,X(T),{\mathscr {U}}_1)$ satisfying Assumptions (LX) and (L2). Since the future payments to the clients, and subsequently the value of the liabilities, depend on the performance of the assets for various insurance products, the value of the liabilities is assumed to be proportional to *v*, which represents the wealth of the insurance company. The term $L_1(T)$ can be used to model a component of the liabilities that is not directly connected to the wealth. This may include both, unhedgeable risks, e.g. mortality risk and hedgeable risks such as interest-rate risk.

#### Example 5

(Performance-linked liabilities with capped benefits and unhedgeable risks (PLUCB)) We specify the model from the previous example further using an affine model. The use of an affine model in this context can also be found in [7]. In this example, we assume that ${\mathscr {U}}_1$ is uniformly distributed on [*a*, *c*], with $a,c \ge 0$, $a<c$. Furthermore, we consider liabilities of the form

PLUCB $$\begin{aligned} L_2(T)=0,\;\;L(T,v) =vL_1(T,X(T),{\mathscr {U}}_1)= v L(0)\left( \beta _1 f(X(T)) + \beta _2 {\mathscr {U}}_1 \right) ,\,L(0) > 0, \end{aligned}$$

with *X* as in Example 1, *f* being a strictly positive function, which is bounded from above by a constant *K* with $0< K < \frac{1}{\beta _1} \left( \frac{1}{\psi _L L(0)} - \beta _2 c \right)$, $\beta _1, \beta _2 \ge 0$, and almost everywhere twice continuously differentiable. These liabilities are a special case of (PLU) and satisfy Assumptions (LX) (see above) and (L2) with $L_1(T)=L(0)(\beta _1f(X(T))+\beta _2{\mathscr {U}}_1)$, $L_2(T)=0$, $k_1=L(0)(\beta _1 K + \beta _2c)$ and $k_2(\omega )=0$ (see also the proof of Corollary 3 for (L2.1)). In addition to the consideration of a general *f*, we also deal with special choices of f:

Choosing *f* as in (7) introduces a positive correlation between the risky asset and $L_1(T)$. In the context of performance-linked liabilities, this leads to liabilities, which are more sensitive to market changes than the wealth process. If we use the function

8 $$\begin{aligned} f(x) =\min \left\{ \frac{1}{x},K\right\} , \end{aligned}$$

this leads to a framework in which the policy holder participates only partially in the performance of the assets. In particular, the liabilities can be written as

$$\begin{aligned} L(T,v)=vL_1(T,X(T),{\mathscr {U}}_1)=L(0)\left( \beta _1\min \left\{ \frac{v}{X(T)},vK\right\} +\beta _2 {\mathscr {U}}_1v\right) \end{aligned}$$

and can therefore be interpreted as a capped relative performance of the insurance company’s wealth compared to an index. In addition, there is an unhedgeable component which can be nicely interpreted in the context of mortality risk: while the first term $\beta _1\min \left\{ \frac{v}{X(T)},vK\right\}$ includes current estimates of the mortality, additional capital $\beta _2{\mathscr {U}}_1v$ must be provided to cover the risk that the mortality changes more than expected in an unfavorable way. In this context, the exact amount of additional capital required is unknown in $t=0$.

#### Example 6

(Performance linked liabilities with capped benefits (PLUCB*)) [16] considers liabilities modeled as geometric Brownian motions which may also include unhedgeable risks. We adapt this model for the liabilities to the context of performance-linked liabilities. We consider more general liabilities of the form

$$\begin{aligned} L_2(T)=0,\;\; L(T,v) := v L_1(T,X(T),{\mathscr {U}}_1), \end{aligned}$$

with

PLUCB* $$\begin{aligned} L_1(T,X(T),{\mathscr {U}}_1) := L(0)f(X(T){\mathscr {U}}_1(T)),\,L(0) > 0, \end{aligned}$$

with X as in Example 1, *f* being a strictly positive function, which is bounded from above by a constant *K* with $0< K < \frac{1}{\psi _L L(0)}$, almost everywhere twice continuously differentiable and ${\mathscr {U}}_1(t)$ given by the SDE

$$\begin{aligned} d{\mathscr {U}}_1(t) = {\mathscr {U}}_1(t) {\hat{\sigma }}_\epsilon dW_\epsilon (t),\,{\mathscr {U}}_1(0)=1, \end{aligned}$$

with constant ${\hat{\sigma }}_\epsilon$ and $W_\epsilon$ being a Brownian motion which is independent of *W*. As in the previous example, these liabilities are a special case of (PLU) and satisfy Assumptions (LX) (see above) and (L2) with $L_1(T)=L(0)f(X(T){\mathscr {U}}_1)$, $L_2(T)=0$, $k_1=L(0) K$ and $k_2(\omega )=0$ (see also the proof of Corollary 5 for (L2.1)). In addition to the consideration of a general *f*, we also deal with special choices of f:

[18] use geometric Brownian motions to model stock prices with a market risk component and an idiosyncratic component. We proceed similarly to model an index and the risk that the actual portfolio of the insurance company deviates from this index. For *X* as in Example 1 representing an index, we interpret $X{\mathscr {U}}_1$ as a fund which uses the index *X* as a benchmark. The SDE of the fund is with Itô’s formula given by

$$\begin{aligned} d(X(t){\mathscr {U}}_1(t))&=X(t){\mathscr {U}}_1(t){\hat{\sigma }}_\epsilon dW_\epsilon (t)+{\mathscr {U}}_1(t)X(t)\left[ {\hat{\mu }}_Xdt+{\hat{\sigma }}_XdW(t)\right] \\&=X(t){\mathscr {U}}_1(t)\left[ {\hat{\mu }}_Xdt+{\hat{\sigma }}_XdW(t)+{\hat{\sigma }}_\epsilon dW_\epsilon (t)\right] , \end{aligned}$$

with ${\hat{\sigma }}_\epsilon$ representing the risk that the portfolio deviates from the index. For *f* as in (8), the value of the liabilities can be written as

$$\begin{aligned} L(T,v)=&\,vL_1(T,X(T))=L(0)\min \left\{ \frac{v}{X(T){\mathscr {U}}_1(T)},vK\right\} . \end{aligned}$$

As in the previous example, the liabilities can be interpreted as a capped relative performance of an asset portfolio, which is, in this case, compared to a fund $X{\mathscr {U}}_1$.

### Surplus optimization framework

#### Optimization problem

We now introduce the portfolio optimization problem for a twice continuously differentiable utility function $U: (0,\infty )\rightarrow {\mathbb {R}}$ with $U'(v)>0$ and $U''(v)<0$ for all $v\in (0,\infty )$. We aim at finding the optimal allocation in the risky asset and the risk-free asset such that the *expected utility of the terminal surplus is maximized*:

$$\begin{aligned} \max _{\pi \in {\varLambda }(v_0)}{\mathbb {E}}\left[ U(S(T))\right] =\max _{\pi \in {\varLambda }(v_0)}{\mathbb {E}}\left[ U(V(T)-\psi _LL(T,V(T)))\right] \qquad (\text {P}_{\text {L}}) \end{aligned}$$

Corresponding to *U*, we define the inverse marginal utility

$$\begin{aligned} I(y):=(U')^{-1}(y),\quad y\in (0,\infty ). \end{aligned}$$

In what follows we always assume that

9 $$\begin{aligned} {\mathbb {E}}\left[ {\tilde{Z}}(T)I(y{\tilde{Z}}(T))\right] <\infty \end{aligned}$$

and

$$\begin{aligned} {\mathbb {E}}\left[ U(I(y{\tilde{Z}}(T))\right] <\infty \end{aligned}$$

for all $$y>0$$.

##### Remark 2

We wish to stress again (now emphasizing the dependence of the wealth *V* on $\pi$) that we focus on the maximization of the expected terminal surplus

$$\begin{aligned} \max _{\pi \in {\varLambda }(v_0)}{\mathbb {E}}\left[ U(V^\pi (T)-\psi _L L(T,V^\pi (T)))\right] \,, \end{aligned}$$

in the presence of liabilities. In contrast, [4] focus on the optimization with fixed-term securities

$$\begin{aligned} \max _{(\psi ,\pi )\in {\varLambda }(v_0)}{\mathbb {E}}\left[ U(V^{(\psi ,\pi )}(T) + \psi F(T))\right] , \end{aligned}$$

where $\psi$ denotes the units invested into the fixed-term security *F*(*T*). For this setting, [4] set up a generalized martingale approach. The following results, namely Lemma 1, Lemma 2 and Theorem 1, can be derived in a similar manner as the corresponding results in [4].

We introduce the random utility functions

$$\begin{aligned} {\bar{U}}(v)={\bar{U}}(v,\omega ):=&\,U\left( v-\psi _LL(T,v)\right) \\ =&\,U(v-\psi _LL(T,v,X(T,\omega ),{\mathscr {U}}_1(\omega ),{\mathscr {U}}_2(\omega ))),\, v\in ({\hat{v}}_0(\omega ),\infty ),\,\omega \in {\varOmega }, \end{aligned}$$

and ${\hat{U}}_{\omega } : ({\hat{v}}_0(\omega ),\infty ) \rightarrow {\mathbb {R}}, \, \omega \in {\varOmega }$, given by

$$\begin{aligned} {\hat{U}}_{\omega }(v) := {\mathbb {E}}\left[ {\bar{U}}(v)\vert {\mathscr {F}}_T\right] = {\mathbb {E}}\left[ U\left( v -\psi _LL(T,v) \right) \big \vert {\mathscr {F}}_T\right] (\omega ). \end{aligned}$$

Note that ${\hat{v}}_0(\omega )\in \left( 0,\frac{\psi _Lk_2(\omega )}{1-\psi _Lk_1}\right]$. Also note that for the case of replicable liabilities, i.e. there are no unhedgeable components ${\mathscr {U}}_1$ and ${\mathscr {U}}_2$, we obviously have

$$\begin{aligned} {\hat{U}}_{\omega }(v) = {\bar{U}}(v,\omega )\,,\quad \omega \in {\varOmega }\,. \end{aligned}$$

The subsequent lemmata are needed for further derivations.

##### Lemma 1

*The random utility function* ${\hat{U}}_{\omega }$ *is differentiable for almost every* $\omega \in {\varOmega }$ *with*

$$\begin{aligned} {\hat{U}}^{\prime }_{\omega }(v) = {\mathbb {E}}\left[ (1-\psi _LL_1(T))U^{\prime }\left( v -\psi _LL(T,v)\right) \big \vert {\mathscr {F}}_T\right] . \end{aligned}$$

Furthermore, ${\hat{U}}^{\prime }_{\omega }$ is strictly decreasing, ${\hat{U}}^{\prime }_{\omega }({\hat{v}}_0(\omega )) \in (0,\infty ]$, ${\hat{U}}^{\prime }_{\omega }(v) > 0$ for all $v > {\hat{v}}_0(\omega )$ and ${\hat{U}}^{\prime }_{\omega }(v) \rightarrow 0$ as $v \rightarrow \infty$.

##### Proof

The proof is provided in the “Appendix”. $\square$

Since ${\hat{U}}_{\omega }$ is differentiable with ${\hat{U}}'_\omega : ({\hat{v}}_0(\omega ),\infty )\rightarrow (0,{\hat{U}}'_\omega ({\hat{v}}_0(\omega )))$, we can define the inverse marginal utility corresponding to ${\hat{U}}_{\omega }$ and we denote it by ${\hat{I}}_\omega :(0,\infty )\rightarrow ({\hat{v}}_0(\omega ),\infty )$, where we set ${\hat{I}}_\omega (y):={\hat{v}}_0(\omega )$ for $y\ge {\hat{U}}'_\omega ({\hat{v}}_0(\omega ))$. Note that, due to the structure of the liabilities, ${\hat{I}}_\omega$ is a deterministic function of *y* and *X*(*T*). Therefore, we also write

$$\begin{aligned} {\hat{I}}_\omega (y)={\mathscr {I}}(y,X(T))={\mathscr {I}}(y,g(X(t),\xi (t,T))). \end{aligned}$$

The following assumption will be used for the derivation of optimal investment strategies.

##### Assumption (LS)

${\mathscr {I}}(y{\tilde{Z}}(t,T),g(x,\xi (t,T)))$ is almost everywhere twice continuously differentiable with respect to both, *x* and *y*. $L_i(T,g(x,\xi (t,T)))),\,i=1,2$ are almost everywhere twice continuously differentiable with respect to *x*.

Furthermore, we define

$$\begin{aligned} H(t,y,x):=&\,{\mathbb {E}}\left[ {\tilde{Z}}(t,T){\hat{I}}_\omega (y{\tilde{Z}}(t,T))|{\mathscr {F}}_t\right] \\ =&\,{\mathbb {E}}\left[ {\tilde{Z}}(t,T){\mathscr {I}}\left( y{\tilde{Z}}(t,T),g(x,\xi (t,T))\right) |{\mathscr {F}}_t\right] \; x,y>0,\,t\in [0,T]. \end{aligned}$$

The following Lemma provides a necessary condition required for the later application of the generalized martingale approach.

##### Lemma 2

*It holds that*

$$\begin{aligned} H(0,y,X(0))= {\mathbb {E}}[{\tilde{Z}}(T){\hat{I}}_{\omega }(y{\tilde{Z}}(T))] < \infty , \quad \text {for all } y > 0. \end{aligned}$$

*Furthermore,* *H*(0, *y*, *X*(0)) *is continuous and strictly monotonically decreasing in* *y*.

##### Proof

The proof is provided in the “Appendix”. $\square$

The following result states that the well-known calculation of the optimal terminal wealth using the martingale approach (see e.g. Theorem 7.6 (p. 114) in [11]) can also be transferred to the case with a random utility function, which is based on the terminal surplus $S(T) = V(T) - \psi _L L(T,V)$.

##### Theorem 1

(Optimal Terminal Wealth) *The optimal terminal wealth for* ($\text {P}_{\text {L}}$) *is given by*

$$\begin{aligned} V^*(T)={\hat{I}}_\omega (Y(v_0) {\tilde{Z}}(T)), \end{aligned}$$

*with* $Y(\cdot )$ *being the inverse of* $H(0,\cdot ,X(0))$. *The optimal terminal surplus is then given by*

$$\begin{aligned} S^*(T):=V^*(T)-\psi _L L(T,V^*(T)). \end{aligned}$$

##### Proof

The proof is provided in the “Appendix”. $\square$

In case of a surplus optimization without performance-linked liabilities, i.e. $L_1(T)\equiv 0$, we have for very unfavorable market developments, i.e. high values of ${\tilde{Z}}(T)$, $V^*(T)={\hat{I}}_\omega (Y(v_0) {\tilde{Z}}(T))={\hat{v}}_0(\omega )=\sup _{{\hat{\omega }}\in {\varOmega }}\psi _LL_2(T,X(T,\omega ),{\mathscr {U}}_2({\hat{\omega }}))$. Thus, the surplus $S^*(T)$ consists only of the difference between the worst case scenario of the unhedgeable risks ${\hat{v}}_0(\omega )$ and the actual realization $\psi _L L_2(T,X(T,\omega ),{\mathscr {U}}_2(\omega ))$.

#### Optimal investment strategy

For the optimal terminal wealth from the previous section, we now deduce the corresponding replicating strategy.

##### Theorem 2

(Optimal Investment Strategy)* Let Assumption (LS) be satisfied. The investment strategy corresponding to the optimal terminal wealth from Theorem* 1*is given by*

$$\begin{aligned} \pi (t)=\frac{1}{\sigma (t)V^{*}(t)}\left[ -H_y\left( t,{\mathscr {Y}}(t),X(t)\right) {\mathscr {Y}}(t)\gamma (t)+H_x\left( t,{\mathscr {Y}}(t),X(t)\right) \sigma _X(t,X(t))\right] , \end{aligned}$$

*with*

$$\begin{aligned} {\mathscr {Y}}(t):=&\,Y(v_0){\tilde{Z}}(t)=Y(v_0)e^{-\int _0^tr(s)+\frac{1}{2}\gamma (s)^2ds-\int _0^t\gamma (s)dW(s)}. \end{aligned}$$

*Furthermore,*

$$\begin{aligned} V^*(t)=&\,H(t,{\mathscr {Y}}(t),X(t)). \end{aligned}$$

##### Proof

The proof is provided in the “Appendix”. $\square$

##### Remark 3

Note that, due to the structure of $\pi$ and *H*, a tractable solution for the optimal investment strategy requires a closed-form representation of ${\hat{I}}_\omega$, which can not be found in general. Later, we present several types of liabilities, for which we can indeed determine ${\hat{I}}_\omega$ in closed form. For replicable liabilities, we find solutions for liabilities which include both, a performance-linked and an index-linked component. We deal with these cases in Sect. 3. In Sect. 4, we consider liabilities with unhedgeable risks.

## Replicable liabilities

In this section, we consider replicable liabilities as introduced in (PILR). We present the general case in Sect. 3.1 and an application with a closed-form solution for the optimal investment strategy in Sect. 3.2. Throughout the whole section, we consider a power utility function with relative risk aversion $b > 0,\,b\ne 1$ of the form

10 $$\begin{aligned} U(v)=\frac{v^{1-b}}{1-b}\,, \end{aligned}$$

to model the preferences of the investor.

### Performance- and index-linked liabilities (PILR)

As a direct consequence of Theorem 2 we obtain:

#### Corollary 1

(Power Utility and Replicable Liabilities (PILR)) *Let Assumption (LS) be satisfied. The optimal terminal wealth for an investor with power utility function* (10) *and liabilities as in* (PILR) *is given by*

$$\begin{aligned} V^*(T) = {\bar{I}}(Y(v_0){\tilde{Z}}(T)) = \frac{1}{1-\psi _LL_1(T)}\left( \left( \frac{Y(v_0){\tilde{Z}}(T)}{1-\psi _LL_1(T)}\right) ^{-\frac{1}{b}} + \psi _L L_2(T)\right) , \end{aligned}$$

*with* ${\bar{I}}$ *denoting the inverse of* ${\bar{U}}'$ *and*

$$\begin{aligned} Y(v_0)=\left( \frac{v_0-{\mathbb {E}}\left[ {\tilde{Z}}(T)\frac{\psi _LL_2(T)}{1-\psi _LL_1(T)}\right] }{{\mathbb {E}}\left[ \left( \frac{{\tilde{Z}}(T)}{1-\psi _LL_1(T)}\right) ^{\frac{b-1}{b}}\right] }\right) ^{-b}. \end{aligned}$$

*Furthermore,*

$$\begin{aligned} V^*(t)=&\,H(t,{\mathscr {Y}}(t),X(t))\\ =&\,{\mathscr {Y}}(t)^{-\frac{1}{b}}{\mathbb {E}}\left[ \left( \frac{{\tilde{Z}}(t,T)}{1-\psi _LL_1(T,g(X(t),\xi (t,T)))}\right) ^{\frac{b-1}{b}}\bigg |{\mathscr {F}}_t\right] \\&+{\mathbb {E}}\left[ {\tilde{Z}}(t,T)\frac{\psi _LL_2(T,g(X(t),\xi (t,T)))}{1-\psi _LL_1(T,g(X(t),\xi (t,T)))}\bigg |{\mathscr {F}}_t\right] \end{aligned}$$

*and the optimal investment strategy is given by*

$$\begin{aligned} \pi ^*(t)= \pi _M(t)+\pi _{PL}(t)+\pi _{IL}(t)+\pi _{mi}(t), \end{aligned}$$

*with the Merton portfolio*

$$\begin{aligned} \pi _M(t)=\frac{\gamma (t)}{b\sigma (t)}, \end{aligned}$$

*performance-linked part*

$$\begin{aligned} \pi _{PL}(t)=&\,\frac{\sigma _X(t,X(t))}{\sigma (t)V^*(t)}{\mathbb {E}}\left[ {\tilde{Z}}(t,T)\left( \frac{1}{b}-1\right) (-\psi _LL_{1,x}(T,X(T)))g_x(X(t),\xi (t,T)))\right. \\&\cdot \left. \left[ 1-\psi _LL_1(T,g(X(t),\xi (t,T)))\right] ^{\frac{1}{b}-2}\left( {\mathscr {Y}}(t){\tilde{Z}}(t,T)\right) ^{-\frac{1}{b}}\bigg |{\mathscr {F}}_t\right] , \end{aligned}$$

*index-linked part*

$$\begin{aligned} \pi _{IL}(t)=&\,\frac{\psi _L}{\sigma (t)V^*(t)} {\mathbb {E}}\left[ {\tilde{Z}}(t,T)\left( \frac{bL_{2,x}(T,X(T)g_x(X(t),\xi (t,T))-\gamma (t)L_2(T,X(T))}{b(1-\psi _LL_1(T,X(T)))}\right) \bigg |{\mathscr {F}}_t\right] \end{aligned}$$

*and a mixed part*

$$\begin{aligned} \pi _{mi}(t)= \frac{\sigma _X(t,X(t))}{\sigma (t)V^*(t)}{\mathbb {E}}\left[ {\tilde{Z}}(t,T)\frac{\psi _LL_2(T,X(T))\psi _LL_{1,x}(T,X(T))g_x(X(t),\xi (t,T)))}{(1-\psi _LL_1(T,X(T)))^2}\bigg |{\mathscr {F}}_t\right] , \end{aligned}$$

*where* $L_{i,x}(T,X(T)),\,i=1,2$ *denote the dereivatives of* $L_i(T,X(T))$ *with respect to the second component.*

#### Proof

The proof is provided in the “Appendix”. $\square$

In this corollary, the portfolios $\pi _{PL}$ (respectively $\pi _{IL}$) are zero when $L_1(T,X(T))$ (respectively $L_2(T,X(T))$) is zero. In each of the two cases, $\pi _{mi}$ is also zero. We will particularly examine these special cases. The case $L_2(X(T))=0$ will be covered in Corollary 4 and the case $L_1(T,X(T))=0$ is treated in the following remark.

#### Remark 4

In case $L_1(T,X(T))=0$ and with Corollary 1,

$$\begin{aligned} v_0={\mathbb {E}}\left[ {\tilde{Z}}(T)V^*(T)\right] =&\,{\mathbb {E}}\left[ {\tilde{Z}}(T) \left( Y(v_0){\tilde{Z}}(T)\right) ^{-\frac{1}{b}}\right] + {\mathbb {E}}\left[ {\tilde{Z}}(T)\psi _L L_2(T)\right] . \end{aligned}$$

The interpretation is that the present value of the optimal terminal surplus is the same as the optimal wealth of an investor with initial capital $v_0-{\mathbb {E}}\left[ {\tilde{Z}}(T)\psi _L L_2(T)\right]$ who maximizes the expected utility of terminal wealth only. Moreover, $\pi _{PL}(t)=\pi _{mi}(t)=0$ and

$$\begin{aligned} \pi ^*(t)=&\,\frac{\gamma (t)}{b\sigma (t)}\cdot \frac{V^*(t)-\psi _L{\mathbb {E}}\left[ {\tilde{Z}}(t,T)L_2(T,X(t))|{\mathscr {F}}_T\right] }{V^*(t)}\\&+\frac{\sigma _X(t,X(t))}{\sigma (t)V^*(t)}\psi _L {\mathbb {E}}\left[ {\tilde{Z}}(t,T)L_{2,x}(T,X(T)g_x(X(t),\xi (t,T))|{\mathscr {F}}_t\right] \\ =&\,\frac{1}{\sigma (t)V^*(t)}\left[ \frac{\gamma (t)}{b}\left( V^*(t)-\psi _L{\mathbb {E}}\left[ {\tilde{Z}}(t,T)L_2(T,X(t))|{\mathscr {F}}_T\right] \right) \right. \\&\left. +\sigma _X(t,X(t))\psi _L {\mathbb {E}}\left[ {\tilde{Z}}(t,T)L_{2,x}(T,X(T)g_x(X(t),\xi (t,T))|{\mathscr {F}}_t\right] \right] . \end{aligned}$$

The first representation shows that the optimal investment strategy can be interpreted as a three-fund separation similar to the one in [3]. In the first term, the Merton portfolio $\frac{\gamma (t)}{\sigma (t)b}$ is scaled by the relative surplus at time *t*. This part is also known as a CPPI strategy (see [2]). The second term represents the liability hedging portfolio and the remaining wealth is invested in the risk-free asset. The second representation shows that the optimal investment strategy can also be interpreted as a CPPI strategy, where the cushion $V^*(t)-\psi _L{\mathbb {E}}\left[ {\tilde{Z}}(t,T)L_2(T,X(t))|{\mathscr {F}}_T\right]$ is adjusted by a part which accounts for the risk of the liabilities. Depending on the structure of the liabilities, this adjustment may be positive or negative.

For this case, i.e. liabilities as in (PILR) with $L_1(T,X(T))=0$, we consider a particular application which allows for a closed-form solution for the investment strategy in the following section.

### Index-linked liabilities with capped maximum benefits (ILRCB)

For the first example with a specific liability model which admits a closed-form solution, we assume constant coefficients $\mu ,\sigma$ and *r* for $P_0$ and *P* and liabilities as in (ILRCB). As a consequence of Theorem 2 , we obtain the following result:

#### Corollary 2

(Index-Linked Liabilities with Capped Maximum Benefits (ILRCB)) *The optimal investment strategy for an investor with power utility function* (10) *and liabilities as in* (ILRCB) *is given by*

$$\begin{aligned} \pi (t)&=\frac{1}{\sigma (t)V^{*}(t)}\left[ \frac{\gamma (t)}{b}\left( V^*(t)-\psi _LL(0)e^{-r(T-t)}\left( K{\varPhi }(d_2(t))+X(t)e^{\left( {\hat{\mu }}_X-{\hat{\sigma }}_X\gamma \right) (T-t)}{\varPhi }(-d_1(t))\right) \right) \right. \\&\quad \left. \vphantom {\left[ \frac{\gamma (t)}{b}\left( V^*(t)-\psi _LL(0)e^{-r(T-t)}\left( K{\varPhi }(d_2(t))+X(t)e^{\left( {\hat{\mu }}_X-{\hat{\sigma }}_X\gamma \right) (T-t)}{\varPhi }(-d_1(t))\right) \right) \right. }+\psi _LL(0)e^{-r(T-t)}e^{\left( {\hat{\mu }}_X-{\hat{\sigma }}_X\gamma \right) (T-t)}{\varPhi }(-d_1(t)){\hat{\sigma }}_XX(t)\right] , \end{aligned}$$

*with*

$$\begin{aligned} d_1(t)=&\,\frac{\log \left( \frac{X(t)}{K}\right) +\left( {\hat{\mu }}_X-{\hat{\sigma }}_X\gamma +\frac{1}{2}{\hat{\sigma }}_X^2\right) (T-t)}{{\hat{\sigma }}_X\sqrt{T-t}},\\ d_2(t)=&\,d_1(t)-{\hat{\sigma }}_X\sqrt{T-t} \end{aligned}$$

*and* ${\varPhi }(x)$ *denotes the cumulative distribution function of a standard normally distributed random variable.*

#### Proof

The proof is provided in the “Appendix”. $\square$

We conclude that this specification of the liability process leads to an optimal investment strategy that can be stated in closed form. Since $\sigma _X>0$, the adjustment $\psi _LL(0)e^{-r(T-t)}e^{\left( {\hat{\mu }}_X-{\hat{\sigma }}_X\gamma \right) (T-t)}{\varPhi }(-d_1(t)){\hat{\sigma }}_XX(t)$ of the investment in the risky asset (see Remark 4) is positive. However, non-hedgeable risks are not included in this liability specification and the resulting investment strategy does not have an impact on the value of the liabilities. We address these extensions in the next section.

## Liabilities with unhedgeable risks

In this section, we derive an optimal strategy for an investor with liabilities that include an asset performance participation under the assumption of a power utility function (10) and liabilities of the form (PLU). As we are not aware of a closed-form solution for ${\hat{I}}_\omega$ in a model with combined index-linked liabilities and unhedgeable risks, we do not consider an index-linked component here.

### Theorem 3

(Terminal Wealth: Performance-Linked Liabilities (PLU)) *The optimal terminal wealth for an investor with power utility function* (10) *and performance-linked liabilities as in* (PLU) *is given by*

$$\begin{aligned} V^*(T) = \left( {\varDelta }_\omega Y(v_0){\tilde{Z}}(T)\right) ^{-\frac{1}{b}}, \end{aligned}$$

*with*

$$\begin{aligned} {\varDelta }_\omega :=&\,\left( {\mathbb {E}}[\left( 1-\psi _LL_1(T)\right) ^{1-b}|{\mathscr {F}}_T]\right) ^{-1} \end{aligned}$$

*and*

$$\begin{aligned} Y(v_0):=&\,\left( \frac{1}{v_0}{\mathbb {E}}\left[ {\tilde{Z}}(T)\left( {\varDelta }_\omega {\tilde{Z}}(T) \right) ^{-\frac{1}{b}}\right] \right) ^{b}. \end{aligned}$$

### Proof

The proof is provided in the “Appendix”. $\square$

The structure of the terminal wealth is similar to the terminal wealth of an investor with power utility and without considering the surplus. The surplus is taken into account by an adjustment factor

$$\begin{aligned} {\varDelta }_\omega ^{-\frac{1}{b}}=\left( {\mathbb {E}}[\left( 1-\psi _LL_1(T)\right) ^{1-b}|{\mathscr {F}}_T]\right) ^{\frac{1}{b}}, \end{aligned}$$

where the term

$$\begin{aligned} 1-\psi _LL_1(T)=\frac{S(T)}{V(T)} \end{aligned}$$

can be interpreted as the terminal surplus relative to the terminal wealth of the asset portfolio. From the structure of ${\varDelta }_\omega$, we can also deduce that the unhedgeable risks (i.e. risks independent of ${\mathscr {F}}_T$) are considered in the optimal terminal wealth (and subsequently in the investment strategy leading to the terminal wealth) only in expectation. The actual realization of the unhedgeable risks only has an impact on the terminal surplus, but not the terminal wealth. However, the hedgeable parts of $L_1(T)$ lead to the following impact of the adjustment factor ${\varDelta }_\omega$: for $b>1$, an $\omega \in {\varOmega }$ resulting in a larger relative surplus leads to a smaller adjustment factor ${\varDelta }_\omega ^{-\frac{1}{b}}$, whereas for $b<1$, a larger relative surplus leads to a larger adjustment factor. This means that the adjustment factor ${\varDelta }_\omega ^{-\frac{1}{b}}$ has a dampening function on the volatility of the terminal wealth for $b>1$, which results from the high risk aversion. On the other hand, a less risk-averse investor with $b<1$ uses a large capital buffer to increase the final wealth. The limit $b\rightarrow 1$ can be interpreted as an investor with logarithmic utility in terms of the relative risk aversion and for such an investor, we have

$$\begin{aligned} {\mathbb {E}}\left[ U(S(T))\right]&={\mathbb {E}}\left[ \log \left( V(T)-\psi _LV(T)L_1(T)\right) \right] \\&={\mathbb {E}}\left[ \log (V(T))\right] +{\mathbb {E}}\left[ (1-\psi _LL_1(T))\right] . \end{aligned}$$

Hence, the solution to ($\text {P}_{\text {L}}$) is independent of $L_1(T)$, the relative surplus does not have an impact on the terminal wealth and therefore, this limit separates the cases $b<1$ and $b>1$.

We can also deduce optimal investment strategies in the setting with performance-linked liabilities. In the following theorem, we consider ${\varDelta }_\omega$ as a function of *X*(*T*), in particular ${\varDelta }_\omega (X(T))={\varDelta }_\omega (g(X(t),\xi (t,T))$.

### Theorem 4

(Investment Strategy: Performance-Linked Liabilities (PLU)) *Let Assumption (LS) be satisfied. The optimal terminal wealth for an investor with power utility function* (10) *and performance-linked liabilities as in* (PLU) *is given by*

$$\begin{aligned} \pi ^*(t)=\frac{\gamma (t)}{b\sigma (t)}+\frac{1}{\sigma (t)V^{*}(t)}H_x\left( t,{\mathscr {Y}}(t),X(t)\right) \sigma _X(t,X(t)), \end{aligned}$$

*with*

$$\begin{aligned} {\mathscr {Y}}(t)=Y(v_0){\tilde{Z}}(t), \end{aligned}$$

$Y(v_0)$ *as in Theorem* 3*and*

$$\begin{aligned} H_x(t,{\mathscr {Y}}(t),X(t))={\mathscr {Y}}(t)^{-\frac{1}{b}}{\mathbb {E}}\left[ {\tilde{Z}}(t,T)^{\frac{b-1}{b}}\frac{\partial }{\partial X(t)}\left( {\varDelta }_\omega (g(X(t),\xi (t,T)))\right) ^{-\frac{1}{b}}|{\mathscr {F}}_t\right] . \end{aligned}$$

*Furthermore,*

$$\begin{aligned} V^*(t)= {\mathbb {E}}\left[ {\tilde{Z}}(t,T)\left( {\varDelta }_\omega \left( g(X(t),\xi (t,T))\right) {\mathscr {Y}}(t){\tilde{Z}}(t,T)\right) ^{-\frac{1}{b}}|{\mathscr {F}}_t\right] . \end{aligned}$$

### Proof

The proof is provided in the “Appendix”. $\square$

In Theorem 4, we observe again a three-fund solution. In contrast to Corollary 1, where the performance seeking part was scaled by the relative surplus at time *t*, it is given by the Merton portfolio $\frac{\gamma (t)}{b\sigma (t)}$. This is due to the fact that we are not considering an index-linked component here ($L_2(T)\equiv 0$). The second term is again the liability hedging portfolio and the remaining wealth is invested in the risk-free asset. In the case of constant coeficients, which will be of particular relevance for our numerical examples, we obtain

$$\begin{aligned}&H_x(t,{\mathscr {Y}}(t),X(t))\\&\quad ={\mathscr {Y}}(t)^{-\frac{1}{b}}{\mathbb {E}}\left[ \left( e^{-\left( r+\frac{1}{2}\gamma ^2\right) (T-t) - \gamma (W(T)-W(t))}\right) ^{\frac{b-1}{b}}\frac{\partial }{\partial X(t)}\left( {\varDelta }_\omega (g(X(t),\xi (t,T)))\right) ^{-\frac{1}{b}}|{\mathscr {F}}_t\right] \\&\quad ={\mathscr {Y}}(t)^{-\frac{1}{b}}e^{\frac{1-b}{b}\left( r+\frac{1}{2}\gamma ^2\right) (T-t)}{\mathbb {E}}\left[ e^{\frac{\gamma (1-b)}{b}(W(T)-W(t))}\frac{\partial }{\partial X(t)}\left( {\varDelta }_\omega (g(X(t),\xi (t,T)))\right) ^{-\frac{1}{b}}|{\mathscr {F}}_t\right] .\\ \end{aligned}$$

### Performance-linked liabilities with capped benefits and unhedgeable risks (PLUCB)

The preceeding theorem allows for the consideration of liability factors $L_1(T)$ as defined in (PLUCB), which are only partially hedgeable. To illustrate that, we use a two-factor model with one factor representing the hedgeable risks and one factor representing the unhedgeable risks. For the liabilities presented here, we can explicitly calculate

$$\begin{aligned} {\varDelta }_\omega ^{-\frac{1}{b}}=\left( {\mathbb {E}}\left[ \left( 1-\psi _LL(0)\left( \beta _1f(X(T))+\beta _2{\mathscr {U}}_1\right) \right) ^{1-b}|{\mathscr {F}}_T\right] \right) ^{\frac{1}{b}} \end{aligned}$$

and $\frac{\partial }{\partial X(t)}{\varDelta }_\omega ^{-\frac{1}{b}}$ as shown in the following corollary.

#### Corollary 3

(Two-Factor Performance-Linked Liabilities (PLUCB)) *The optimal terminal wealth for an investor with power utility function* (10) *and performance-linked liabilities as in* (PLUCB) *is given by Theorem* 3*and the optimal investment strategy is given by Theorem* 4*with*

$$\begin{aligned} {\varDelta }_\omega ^{-\frac{1}{b}}&= \left( \tfrac{\left( 1- \psi _L L(0)\left( \beta _1 f(X(T)) + \beta _2 a \right) \right) ^{2-b} - \left( 1- \psi _L L(0)\left( \beta _1 f(X(T)) + \beta _2 c \right) \right) ^{2-b}}{(c-a) (\psi _L L(0)\beta _2) (2-b)}\right) ^{\frac{1}{b}}\, \end{aligned}$$

*and*

$$\begin{aligned} \frac{\partial }{\partial X(t)}{\varDelta }_\omega ^{-\frac{1}{b}} =&\,\frac{1}{b}\left( \frac{-\psi _LL(0)\beta _1f'(X(T))g_x(X(t),\xi (t,T))}{\left( (c-a) \left( \psi _L L(0)\beta _2 \right) (2-b)\right) ^{\frac{1}{b}}}\right) \left[ \left( 1-\psi _LL(0)\left( \beta _1f(X(T))+\beta _2a\right) \right) ^{1-b}\right. \\&\left. -\left( 1-\psi _LL(0)\left( \beta _1f(X(T))+\beta _2c\right) \right) ^{1-b} \right] (2-b)\cdot \\&\cdot \left[ \left( 1- \psi _L L(0)\left( \beta _1 f(X(T)) + \beta _2 a \right) \right) ^{2-b}\right. \\&-\left. \left( 1- \psi _L L(0)\left( \beta _1 f(X(T)) + \beta _2 c \right) \right) ^{2-b}\right] ^{\frac{1}{b}-1} \end{aligned}$$

*for* $b\ne 2$. *For* $b=2$, *we have*

$$\begin{aligned} {\varDelta }_\omega ^{-\frac{1}{b}}=\left( \frac{1}{(c-a)(\psi _L L(0)\beta _2 )}\log \left( \frac{1 - \psi _L L(0)\left( \beta _1 f(X(T)) + \beta _2 a \right) }{ 1- \psi _L L(0) \left( \beta _1 f(X(T)) + \beta _2c \right) }\right) \right) ^{\frac{1}{b}} \end{aligned}$$

*and*

$$\begin{aligned} \frac{\partial }{\partial X(t)}{\varDelta }_\omega ^{-\frac{1}{b}}&=\frac{-\psi _LL(0)\beta _1f'(X(T))g_x(X(t),\xi (t,T))}{b\left( (c-a)(\psi _L L(0)\beta _2 )\right) ^{\frac{1}{b}}}\left( \log \left( \frac{1 - \psi _L L(0)\left( \beta _1 f(X(T)) + \beta _2 a \right) }{ 1- \psi _L L(0) \left( \beta _1 f(X(T)) + \beta _2 c \right) }\right) \right) ^{\frac{1}{b}-1}\\&\quad \cdot \left( \frac{1}{1 - \psi _L L(0)\left( \beta _1 f(X(T)) + \beta _2 a \right) }-\frac{1}{1 - \psi _L L(0)\left( \beta _1 f(X(T)) + \beta _2 c \right) }\right) . \end{aligned}$$

#### Proof

The proof is provided in the “Appendix”. $\square$

With ${\mathscr {U}}_1$ being independent of ${\mathscr {F}}_T$ and *X*(*T*) being ${\mathscr {F}}_T$-measurable, the distortion factor ${\varDelta }_\omega ^{-\frac{1}{b}}$ is expressed in terms of an expected value of a function of ${\mathscr {U}}_1$ and the actual realization of the unhedgeable risks does not influence the optimal investment strategy .

#### Remark 5

For *f* as in (7), we have $f'(x)=1$ for $x<K$ and $f'(x)=0$ for $x>K$. This property together with the fact that *f* is constant for $x\ge K$ simplifies the numerical calculation of the expectation from Theorem 4. The case of *f* from (8) is very similar. The same holds true for the two subsequent examples in this section.

In the case $\beta _2=0$, i.e. if there are no unhedgeable risks, the optimal investment strategy simplifies and is given by the following corollary. We denote this type of libilities by (PLRCB).

#### Corollary 4

(Replicable Performance-Linked Liabilities with Capped Benefits (PLRCB)) *The optimal terminal wealth for an investor with power utility function* (10) *and replicable performance-linked liabilities as in* (PLUCB) *with* $\beta _2=0$ *is given by Theorem* 3*and the optimal investment strategy is given by Theorem* 4*with*

$$\begin{aligned} {\varDelta }_\omega ^{-\frac{1}{b}}&= \left( 1-\psi _LL(0)\beta _1f(X(T))\right) ^{\frac{1}{b}-1} \end{aligned}$$

*and*

$$\begin{aligned} \frac{\partial }{\partial X(t)}{\varDelta }_\omega ^{-\frac{1}{b}}=\left( 1-\psi _LL(0)\beta _1 f(X(T))\right) ^{\frac{1}{b}-2} \left( \frac{1}{b}-1\right) \left( -\psi _LL(0)\beta _1f'(X(T))\right) g_x(X(t),\xi (t,T)). \end{aligned}$$

#### Proof

The proof is provided in the “Appendix”. $\square$

It is clearly visible in this example that the factor ${\varDelta }_\omega ^{-\frac{1}{b}}$ only depends on the relative surplus and the risk aversion. As described earlier, Corollary 4 describes a special case of Corollary 1 (with $L_2(T,X(T))=0$).

### Liabilities driven by geometric brownian motion

In this section, we consider the liability model (PLUCB*).

#### Remark 6

In the case of completely hedgeable liabilities, i.e. ${\hat{\sigma }}_I=0$, the liabilities correspond to the setting from Corollary 4 for the choice $\beta _1=1$.

We examine the optimal terminal wealth and optimal investment strategy, in particular

$$\begin{aligned} {\varDelta }_\omega ^{-\frac{1}{b}}=\left( {\mathbb {E}}\left[ \left( 1-\psi _LL(0)f(X(T){\mathscr {U}}_1(T))\right) ^{1-b}\Big \vert {\mathscr {F}}_T\right] \right) ^{\frac{1}{b}} \end{aligned}$$

in the following corollary.

#### Corollary 5

(Liabilities Driven by Geometric Brownian Motion (PLUCB*)) *The optimal terminal wealth for an investor with power utility function* (10) *and performance-linked liabilities as in* (PLUCB*) *is given by Theorem* 3*and the optimal investment strategy is given by Theorem* 4*with*

$$\begin{aligned} \frac{1}{{\varDelta }_\omega }=&\, \int _{-\infty }^{\infty } \left( 1 - \psi _L L(0) f\left( X(T) e^{ - \frac{1}{2} {\hat{\sigma }}_I^2 T + {\hat{\sigma }}_I \sqrt{T} u } \right) \right) ^{1-b} \phi (u)du \end{aligned}$$

*and*

$$\begin{aligned} \frac{\partial }{\partial X(t)}{\varDelta }_\omega ^{-\frac{1}{b}}&=\frac{1}{b}\left( \int _{-\infty }^{\infty } \left( 1 - \psi _L L(0) f\left( g(X(t),\xi (t,T)) e^{ - \frac{1}{2} {\hat{\sigma }}_I^2 T + {\hat{\sigma }}_I \sqrt{T} u } \right) \right) ^{1-b} \phi (u)du\right) ^{\frac{1}{b}-1}\\&\quad \cdot \int _{-\infty }^{\infty } (1-b)\left( 1 - \psi _L L(0) f\left( g(X(t),\xi (t,T)) e^{ - \frac{1}{2} {\hat{\sigma }}_I^2 T + {\hat{\sigma }}_I \sqrt{T} u } \right) \right) ^{-b} \\&\quad \cdot \left( -\psi _LL(0)f'\left( g(X(t),\xi (t,T)) e^{ - \frac{1}{2} {\hat{\sigma }}_I^2 T + {\hat{\sigma }}_I \sqrt{T} u }\right) \right) \\&\quad \cdot g_x(X(t),\xi (t,T)) e^{ - \frac{1}{2} {\hat{\sigma }}_I^2 T + {\hat{\sigma }}_I \sqrt{T} u }\phi (u)du. \end{aligned}$$

#### Proof

The proof is provided in the “Appendix”. $\square$

## Comparison of optimal investment strategies

In this section, we use the different derived results to assess the impact of the liabilities, the performance participation and the unhedgeable risks on the investment strategy. In particular, we use the liability models specified in Examples 3 (ILRCB), 5 (PLUCB), 6 (PLUCB*) and Corollary 4 (PLRCB) as well as a strategy with mixed, replicable liabilities. In Sect. 5.1, we analyze the influence of general parameters on the optimal investment strategy. Compared to the Merton portfolio $\frac{\mu -r}{b\sigma ^2}$, the replicable liabilities from Corollary 2 (Example 3 (ILRCB)) illustrate the influence of the replicable, index-linked liabilities with capped benefits. On the other hand, compared to the results from Corollary 4 (performance-linked, replicable (PLRCB), this setting also serves as a reference point for the assessment of the impact of the performance participation with replicable liabilities. In addition, we consider replicable liabilities as in Corollary 1 (mixed liabilities), with liabilities consisting of an equally-weighted average of the replicable performance-linked and index-linked type. In Sect. 5.2, the impact of the unhedgeable component in the liability risk is studied when comparing the results from Corollary 3 (performance-linked liabilities in a two-factor model, liabilities as in Example 5 (PLUCB)) and Corollary 4 (performance-linked, replicable (PLRCB)). Finally, comparing the results from Corollary 3 (performance-linked liabilities in a two-factor model (PLUCB)) and Corollary 5 (performance-linked liabilities, setting as in Example 6 (PLUCB*)), we compare the influence of the different types of models with unhedgeable risks for the liabilities.

For these comparisons, we compute the optimal allocation in the risky asset in $t=0$ numerically. Unless otherwise mentioned, we consider an investor with power utility function with $b=2$ and a risky asset representing equity with $\mu =0.06$ and $\sigma =0.3$. Furthermore, we choose $r=0.01$, $T=10$, $\psi _L=1$, $V(0)=1$ and $L(0)=0.5$. X is always modeled as in Example 1 with ${\hat{\mu }}_X=0$, ${\hat{\sigma }}_X=0.1$. For the specific parameters of the liability models, we choose $K=1.7$, $\beta _1=\beta _2=0.5$, $a=0$, $c=0.1$, and ${\hat{\sigma }}_I=0.48$. To ensure comparibility of the models, ${\hat{\sigma }}_I$ is chosen such that the optimal allocations of both models with unhedgeable risks, i.e. the models from Corollary 3 (PLUCB) and Corollary 5 (PLUCB*), match for the setting in Sect. 5.2, whereas *L*(0) and *K* are chosen such that the conditions for Corollaries 3, 4 and 5 hold. Due to the typical properties of insurance products, liabilities which are partially linked to the development of an index or the portfolio of the insurance company itself are often most interesting. For *f* as in (7), the index-linked liabilities exhibit such a behavior. However, the performance-linked liabilities are stronger linked to the development of the risky asset than the asset portfolio itself. Therefore, we use *f* from (7) only to analyze the optimal investment strategy with index-linked liabilities and to illustrate the impact of the performance participation in the first part. In the second part, we choose *f* from (8) to get a more realistic assessment of the performance-linked liabilities. In this setting, we compare the different models for the performance-linked liabilities and analyze the impact of unhedgeable risks.

### Impact of the type of the liabilities

**Fig. 1:**
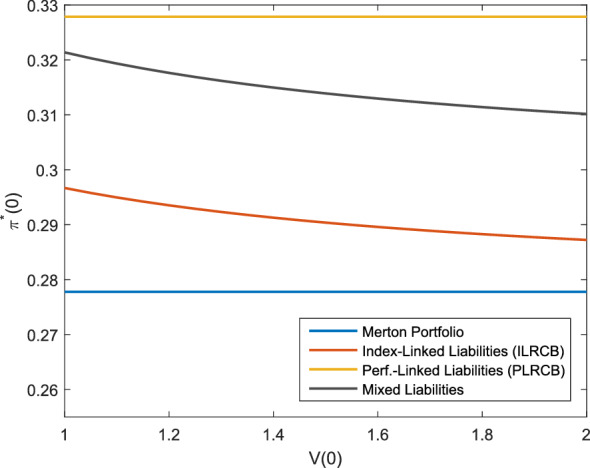
Optimal investment strategy depending on the initial wealth, *f* as in (7)

**Fig. 2:**
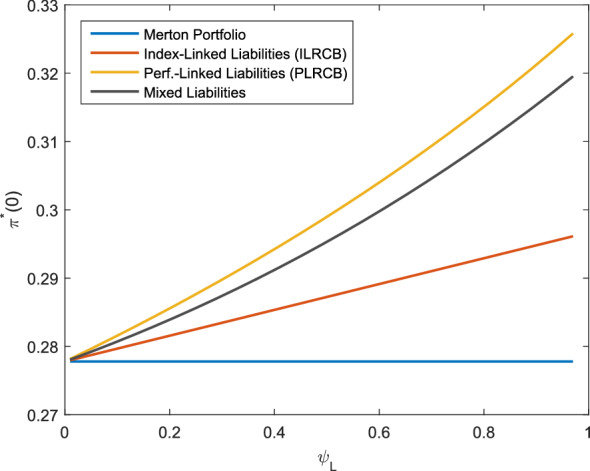
Optimal investment strategy depending on $\psi _L$, *f* as in (7)

In Fig. 1, we see that the optimal allocation in the risky asset is independent of the initial wealth for the model with performance-linked liabilities (PLRCB) as both, the assets and liabilities, increase with *V*(0), whereas the allocation is decreasing in the initial wealth for the model with index-linked liabilities (ILRCB) as a lower initial wealth requires a hedge of the liability risk which makes up a larger part of the portfolio. As *f*(*X*(*T*)) is positively correlated to the asset portfolio here, the higher investment for the index-linked and performance-linked liabilities compared to the Merton portfolio can be interpreted as an additional effort to hedge the liabilities.

The level to which the liabilities are considered influences the optimal investment strategy as Fig. 2 shows. As $\psi _L\rightarrow 0$, the extent to which the liabilities are considered is decreasing and the strategies converge to the Merton portfolio. Again, we observe that the performance-linked liabilities lead to the highest allocation in the risky asset. Similar to the observation in Fig. 1, the positive correlation of *X*(*T*) and the asset portfolio leads for the index-linked and performance-linked liabilities to a larger allocation as the surplus decreases, which can be interpreted as an additional effort to hedge the liabilities.

**Fig. 3:**
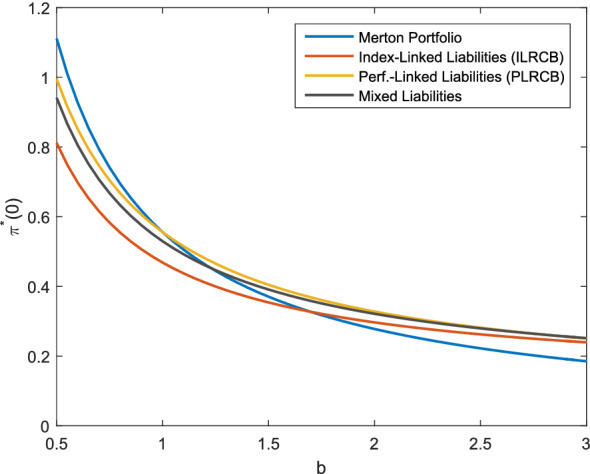
Optimal investment strategy depending on *b*, *f* as in (7)

To examine the effect of the risk aversion, Fig. 3 shows the optimal allocation dependening on *b*. The allocation in the risky asset is decreasing with a higher level of risk aversion in all cases. However, the allocation in the performance-linked case is higher than the allocation of the Merton portfolio for $b>1$ and lower than the Merton portfolio for $b<1$. The higher allocation of the investor being more risk-averse compared to the Merton portfolio can again be interpreted as an additional attempt to hedge the liability risk inherent in $L_1(t)$. As described before, the limit $b\rightarrow 1$ can be interpreted as an investor with logarithmic utility and the optimal investment strategy is independent of $L_1(T)$ for such an investor. Therefore, the performance-linked investment strategies converge to the Merton portfolio as $b\rightarrow 1$. For the index-linked liabilities, the difference of the allocation between a high risk aversion and a low risk aversion is smaller. The reason is that the investor having index-linked liabilities hedges the liabilities regardless of the risk aversion. Only the investment of the remaining wealth is subject to the risk aversion (see the explanation after Corollary 1). In Figs. 1, 2 and 3, the optimal allocation for the mixed liabilities is always between the allocation of the performance-linked liabilities and the index-linked liabilities. To summarize, we see that in the case of index-linked or performance-linked liabilities, an investor invests considerably more in the risky asset than the investor without performance-linked liabilities. Furthermore, we observe that if the surplus is lower or the investor is more risk-averse, additional efforts are made to hedge the liabilities in the case of performance-linked liabilities. The different liability models show that a close link to the insurance company’s portfolio value leads to higher allocations in the risky asset, which should be considered for the product design if, e.g., a high share of risky assets is desired for long-term products. Furthermore, the larger difference in the allocation in Fig. 3 for the performance-linked liabilities compared to the index-linked liabilities shows that in case of performance-linked liabilities, the allocation is more sensitive to *b*, so the insurance company’s risk aversion is more important to the policy holder. This can be explained by the fact that the insurance company’s risk aversion does not have an impact on the performance of the index underlying the index-linked product. However, if the policy holder participates in the insurance company’s asset portfolio, the performance of the policy is directly influenced by the insurance company’s risk aversion. As a consequence, the risk aversion of the insurance company and the policy holder should be consistent for performance-linked products.

**Fig. 4:**
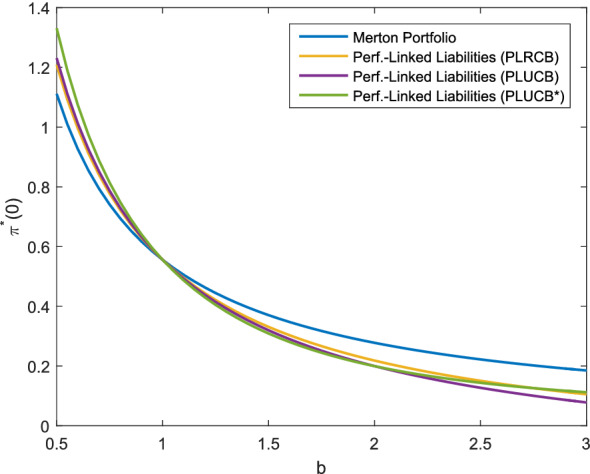
Optimal investment strategy depending on *b*, *f* as in (8)

### Comparison of investment strategies with performance-linked liabilities and non-hedgeable risks

**Fig. 5:**
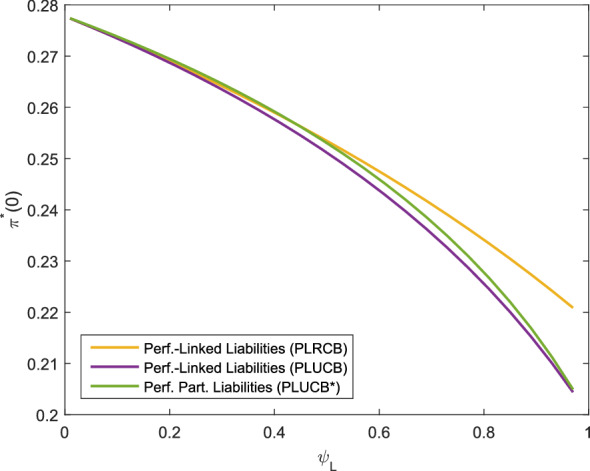
Optimal investment strategy depending on $\psi _L$, *f* as in (8)

**Fig. 6:**
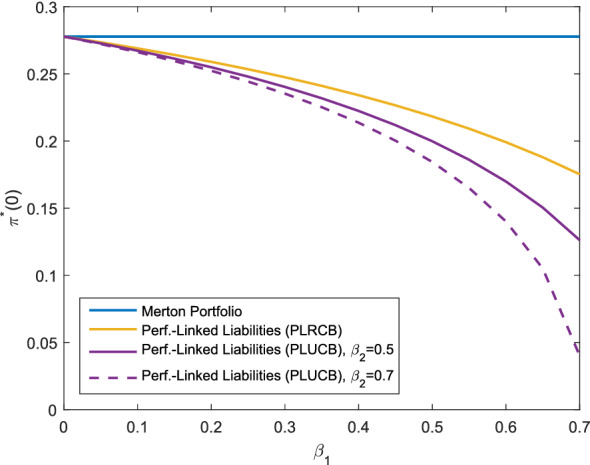
Optimal investment strategy depending on $\beta _1$, *f* as in (8)

In this section, we compare the settings for performance-linked liabilities (Corollaries 4 (PLRCB), 3 (PLUCB) and 5 (PLUCB*)). In particular, we analyze the impact of unhedgeable risks on the optimal investment strategy. For a more realistic model of the liabilities, we use *f* from (8) in this section. By the choice of the parameters, both cases with performance-linked liabilities and additional unhedgeable risks have the same allocation in the base case with the parameters as described above. It is observable from Fig. 4 that for $b>1$, performance-linked liabilities without unhedgeable risks (PLRCB) admit a higher allocation compared to the corresponding liability model with unhedgeable risks (PLUCB) as no buffer for these risks must be provided. For an increasing portion in $\psi _L$, i.e. as the weight of the liabilities increases and the surplus decreases, the allocation in the risky asset is decreasing. This is in contrast to the results presented in Fig. 3, where the difference occurs as *f*(*X*(*T*)) is negatively correlated to the risky asset in the setting of Fig. 4. Moreover, we notice that the allocation in the risky asset for the liabilities (PLUCB*) is slightly higher than the allocation in the the liability model (PLUCB) for $\psi _L\in (0,1)$, while both allocations are the same for $\psi _L=1$ (by construction of the parameter set) and for $\psi _L=0$ (as the liabilities are not at all taken into account). The weight for the consideration of the hedgeable part of the liability risk in the liability model (PLUCB), represented by $\beta _1$, varies in Fig. 6 and is shown for different levels of $\beta _2$, $\beta _2=0.7$, $\beta _2=0.5$ and $\beta _2=0$ (replicable performance-linked liabilities). We see that for larger values of $\beta _1$, the optimal allocation decreases as well as for larger values of $\beta _2$, so an increase in the liability risk leads to a decrease in the allocation. Independent of the level of $\beta _2$, the optimal investment strategy converges to the Merton portfolio as $\beta _1\rightarrow 0$. This can be explained by the observation that for $\beta _1=0$ and any $\beta _2$, the surplus can be represented as a product of the portfolio value and the relative surplus $1-\psi _LL_1(t)$, with $L_1(t)$ being independent of the portfolio value. In summary, Figs. 4, 5 and 6 show that unhedgeable risks such as additional mortality risks lead to a reduction in the risky asset allocation. The effect increases as the risk aversion increases (Fig. 4), the surplus decreases (stronger consideration of the liabilities, Fig. 5) or the portion of the unhedgeable risks increases (Fig. 6). For the design of products, this means that the policy holder suffers form the insurance company’s unhegeable risks through a lower allocation in risky assets, in particular in case the insurance company has little own funds or is very risk-averse.

## Conclusion

In this article, we establish a dynamic optimization framework in continuous time for terminal surplus optimization, which also includes the possibility of index-linked and performance-participating liabilities and liabilities which are not completely hedgeable. In general settings, we derive results for the optimal terminal wealth and the optimal investment strategy. In specific settings, we obtain closed-form solutions for different types of liability models, motivated by several ways to model liabilities from the existing literature. These settings include performance-linked liabilties and index-linked liabilities. Among these models, we study hedgeable liabilities and two models with performance-linked liabilities and unhedgeable risk components. For the different types of specific liability models, we compare the impact of performance participation, unhedgeable risk components, degree of consideration of the liabilities and risk aversion of the investor. We find that performance- or index-linked liabilities allow for a substantial increase in the allocation in the risky asset compared to the Merton portfolio. Index-linked liabilities are less sensitive with respect to the level of consideration of the liabilities than performance-linked liabilities. We also illustrate the convergence of the optimal investment strategy within a model with both hedgeable and unhedgeable risk components to the allocation of a model with only hedgeable risk components and to the Merton portfolio as the influence of the corresponding components is reduced. Moreover, we identify the increasing attempt to hedge the liability risks in the models with performance-linked liabilities for an increasing level of risk aversion. We also find that a close link between the liabilities and wealth leads to a high risky investment whereas unhedgeable risks lead to a reduction in the allocation in the risky investment. Consequently, if a high expected return for the policy holder is aimed at, the performance of insurance products should be closely connected to the company’s wealth and the product should be designed in a way such that unhedgeable risks are reduced as much as possible. Finally, the risk aversion of the insurance company has a larger impact on performance-linked products compared to index-linked products. Thus, for performance-linked products, the policy holder and insurance company need to have consistent risk aversions.

## Appendix

### Proof of Lemma 1

For $\omega \in {\varOmega }$, ${\hat{v}}>{\hat{v}}_0(\omega )$ arbitrary but fixed and $\epsilon$ small enough, we have

$$\begin{aligned} 0&< \frac{\partial }{\partial v}U(v-\psi _LL(T,v))\\&=\frac{\partial }{\partial v}U(v-\psi _L(vL_1(T)+L_2(T)))\\&=(1-\psi _LL_1(T))U'((1-\psi _LL_1(T))v-\psi _LL_2(T))\\&\le U'((1-\psi _LL_1(X(T,\omega ),{\mathscr {U}}_1({\hat{\omega }}_1))({\hat{v}}-\epsilon )-\psi _LL_2(X(T,\omega ),{\mathscr {U}}({\hat{\omega }}_2))) \end{aligned}$$

for all $v\in ({\hat{v}}-\epsilon ,{\hat{v}}+\epsilon )$. Thus, given ${\mathscr {F}}_T$, there is a constant upper bound for $\frac{\partial }{\partial v}U(v -\psi _LL(T,v))$ and differentiation and integration at ${\hat{v}}$ can be interchanged by dominated convergence. The other properties follow by the characteristics of $U^{\prime }$ as well as ${\hat{v}}_0$. $\square$

### Proof of Lemma 2

We have for $v>{\hat{v}}_0(\omega )$ by dominated convergence as in the proof of Lemma 1 and as $U'$ is strictly monotonically decreasing

$$\begin{aligned} {\hat{U}}_{\omega }^{\prime }(v)&=\, {\mathbb {E}}\left[ \frac{\partial }{\partial v}U\left( v-\psi _LL(T,v)\right) \big \vert {\mathscr {F}}_T\right] \\&=\,{\mathbb {E}}\left[ (1-\psi _LL_1(T))U'((1-\psi _LL_1(T))v-\psi _LL_2(T))\big \vert {\mathscr {F}}_T\right] \\&\le U^{\prime }\left( (1-\psi _LL_1(X(T,\omega ),{\mathscr {U}}_1({\hat{\omega }}_1)))v-\psi _LL_2(X(T,\omega ),{\mathscr {U}}_2({\hat{\omega }}_2))\right) \\&=: M_\omega (v) \end{aligned}$$

for a.e. $\omega \in {\varOmega }$. Note that $M_\omega (v)$ is continuous and strictly monotonically decreasing and therefore its inverse

$$\begin{aligned} I^M_\omega (y)={\left\{ \begin{array}{ll}(M_\omega )^{-1}(y),&{}\;\; 0<y<{\hat{U}}'({\hat{v}}_0(\omega ))\\ {\hat{v}}_0(\omega ),&{}\;\;\text {else}\end{array}\right. } \end{aligned}$$

is monotonically decreasing as well. Then, it follows for all $0<y<{\hat{U}}'_\omega ({\hat{v}}_0(\omega ))$

$$\begin{aligned} y = {\hat{U}}_{\omega }^{\prime }\left( {\hat{I}}_\omega (y)\right) \le M_\omega \left( {\hat{I}}_\omega (y)\right) . \end{aligned}$$

By the monotonicity of $I^M_\omega (y)$, we have

$$\begin{aligned} I^M_\omega (y) \ge I^M_\omega \left( M_\omega \left( {\hat{I}}_\omega (y)\right) \right) = {\hat{I}}_\omega (y). \end{aligned}$$

Since both sides are equal for $y>{\hat{U}}_\omega '({\hat{v}}_0(\omega ))$, we learn that ${\hat{I}}_{\omega }(y) \le I_\omega ^M(y)$ for almost every $\omega \in {\varOmega }$. As $L_i(X(T,\omega ),{\mathscr {U}}_i({\hat{\omega }}_i))$ are ${\mathscr {F}}_T$-measurable for $i=1,2$ and due to the boundedness conditions (L2.1) and (L2.2),

$$\begin{aligned} I^M_\omega (y)&\le \max \left\{ \frac{1}{1-\psi _LL_1(X(T,\omega ),{\mathscr {U}}_1({\hat{\omega }}_1))}\left( I\left( y\right) + \psi _L L_2(X(T,\omega ),{\mathscr {U}}_2({\hat{\omega }}_2))\right) ;{\hat{v}}_0(\omega )\right\} \\&\le \max \left\{ \frac{1}{1-\psi _Lk_1}\left( I(y)+\psi _Lk_2(\omega )\right) ;{\hat{v}}_0(\omega )\right\} . \end{aligned}$$

The statement follows with (9) and the continuity and monotonicity of *H*(0, *y*, *X*(0)) follow with Lemma 1. $\square$

### Proof of Theorem 1

We adapt the proof of Theorem 3.3 in [4] (see also [19]) to our setting, in particular to the different continuation of ${\hat{I}}_\omega$.

#### Lemma 3

(Young’s Inequality) *Let* $v>{\hat{v}}_0(\omega )$, $y>0$. *Then,*

$$\begin{aligned} {\hat{U}}_\omega (v)\le {\hat{U}}_\omega ({\hat{I}}_\omega (y))+y\left( v-{\hat{I}}_\omega (y)\right) {\mathbb {Q}}\text {-a.s.} \end{aligned}$$

#### Proof

As *U* is concave and ${\hat{U}}_\omega$ is ${\mathbb {Q}}$-a.s. differentiable as stated in Lemma 1, we have for $v,v_1>{\hat{v}}_0(\omega )$

11 $$\begin{aligned} {\hat{U}}_\omega (v)\le {\hat{U}}_\omega (v_1)+{\hat{U}}'_\omega (v_1)\left( v-v_1\right) . \end{aligned}$$

In the following, we use (11) and set $v_1={\hat{I}}_\omega (y)$. If $y<{\hat{U}}'_\omega ({\hat{v}}_0(\omega ))$, then ${\hat{U}}'_\omega ({\hat{I}}_\omega (y))=y$ and

$$\begin{aligned} {\hat{U}}_\omega (v)\le {\hat{U}}_\omega ({\hat{I}}_\omega (y))+y\left( v-{\hat{I}}_\omega (y)\right) . \end{aligned}$$

If $$y>{\hat{U}}'_\omega ({\hat{v}}_0(\omega ))$$, $${\hat{I}}_\omega (y)={\hat{v}}_0(\omega )$$ and

$$\begin{aligned} {\hat{U}}_\omega (v)&\le {\hat{U}}_\omega ({\hat{I}}_\omega (y))+{\hat{U}}'_\omega ({\hat{I}}_\omega (y))\left( v-{\hat{I}}_\omega (y)\right) \\&= {\hat{U}}_\omega ({\hat{I}}_\omega (y))+{\hat{U}}'_\omega ({\hat{v}}_0(\omega ))\left( v-{\hat{I}}_\omega (y)\right) \\&\le {\hat{U}}_\omega ({\hat{I}}_\omega (y))+y\left( v-{\hat{I}}_\omega (y)\right) \end{aligned}$$

since $v>{\hat{v}}_0(\omega )={\hat{I}}_\omega (y)$ by assumption. $\square$

#### Proof

We consider the set

$$\begin{aligned} {\mathscr {V}}:=\left\{ V {\mathscr {F}}_T\text {-measurable:}\,V\ge {\hat{v}}_0,\, {\mathbb {E}}\left[ {\hat{U}}_\omega (V)^-\right] <\infty ,\,{\mathbb {E}}\left[ {\tilde{Z}}(T)V\right] \le v_0\right\} . \end{aligned}$$

By construction of our financial market, ${\mathscr {V}}$ is the set of all payoffs which can be replicated with an initial wealth of not more than $v_0$ and which cover the value of the liabilities in *T* for every realization of the unhedgeable risks. In the following, we show that $V^*(T):={\hat{I}}_\omega (Y(v_0){\tilde{Z}}(T))$ is the optimal terminal wealth. We observe that $V^*(T)\ge {\hat{v}}_0$ by the definition of ${\hat{I}}_\omega$, $V^*(T)$ is ${\mathscr {F}}_T$-measurable and ${\mathbb {E}}\left[ {\tilde{Z}}(T)V^*(T)\right] =v_0$ by the definition of $Y(v_0)$. Furthermore, with arbitrary $v=V\in {\mathscr {V}}$ and $y=Y(v_0){\tilde{Z}}(T)$, Young’s inequality (see Lemma 3) reads

12 $$\begin{aligned} {\hat{U}}_\omega (V^*(T))&={\hat{U}}_\omega ({\hat{I}}_\omega (Y(v_0){\tilde{Z}}(T)))\nonumber \\&\ge {\hat{U}}_\omega (V)+Y(v_0){\tilde{Z}}(T)\left( {\hat{I}}_\omega (Y(v_0){\tilde{Z}}(T))-V\right) \nonumber \\&={\hat{U}}_\omega (V)+Y(v_0){\tilde{Z}}(T)\left( V^*(T)-V\right) . \end{aligned}$$

Therefore,

$$\begin{aligned} {\hat{U}}_\omega (V^*(T))^- \le {\hat{U}}_\omega (V)^-+Y(v_0){\tilde{Z}}(T)\left( V^*(T)+V\right) \end{aligned}$$

and thus

$$\begin{aligned} {\mathbb {E}}\left[ {\hat{U}}_\omega (V^*(T))^-\right] \le {\mathbb {E}}\left[ {\hat{U}}_\omega (V)^-\right] +Y(v_0)\left( {\mathbb {E}}\left[ {\tilde{Z}}(T)V^*(T)\right] +{\mathbb {E}}\left[ {\tilde{Z}}(T)V\right] \right) <\infty \end{aligned}$$

as $V\in {\mathscr {V}}$ and ${\mathbb {E}}\left[ {\tilde{Z}}(T)V^*(T)\right] =v_0<\infty$. Consequently, $V^*(T)\in {\mathscr {V}}$. Applying (12) again and taking the expactation on both sides of this inequality leads to

$$\begin{aligned} {\mathbb {E}}[{\hat{U}}_\omega (V^*(T))]\ge {\mathbb {E}}[{\hat{U}}_\omega (V)]+Y(v_0)\left( v_0-{\mathbb {E}}[{\tilde{Z}}(T)V]\right) \ge {\mathbb {E}}[{\hat{U}}_\omega (V)], \end{aligned}$$

since ${\mathbb {E}}[{\tilde{Z}}(T)V^*(T)]=v_0$ and ${\mathbb {E}}[{\tilde{Z}}(T)V]\le v_0$. Since

$$\begin{aligned} {\mathbb {E}}\left[ {\hat{U}}_\omega (V)\right] =&\,{\mathbb {E}}\left[ {\mathbb {E}}\left[ U(V-\psi _LL(T,V))|{\mathscr {F}}_T\right] \right] \\ =&\,{\mathbb {E}}\left[ U(V-\psi _LL(T,V))\right] \end{aligned}$$

for all $V\in {\mathbb {V}}$, we see that

$$\begin{aligned} V^*(T)=\arg \max _{V\in {\mathscr {V}}}{\mathbb {E}}\left[ U(V-\psi _LL(T,V))\right] . \end{aligned}$$

$$\square $$

### Proof of Theorem 2

Using Itô’s formula, the SDE of ${\mathscr {Y}}$ can be written as

$$\begin{aligned} d{\mathscr {Y}}(t)=&\,{\mathscr {Y}}(t)\left[ -r(t)ds-\gamma (t)dW(t)\right] . \end{aligned}$$

By Theorem 1,

$$\begin{aligned} V^*(T)={\hat{I}}_\omega (Y(v_0){\tilde{Z}}(T))={\hat{I}}_\omega ({\mathscr {Y}}(T)). \end{aligned}$$

We thus have with Assumption (LX)

$$\begin{aligned} V^*(t)=&\,{\mathbb {E}}\left[ {\tilde{Z}}(t,T){\hat{I}}_\omega \left( {\mathscr {Y}}(T)\right) |{\mathscr {F}}_t\right] \\ =&\,{\mathbb {E}}\left[ {\tilde{Z}}(t,T){\mathscr {I}}\left( {\mathscr {Y}}(T),X(T)\right) |{\mathscr {F}}_t\right] \\ =&\,{\mathbb {E}}\left[ {\tilde{Z}}(t,T){\mathscr {I}}\left( {\mathscr {Y}}(t){\tilde{Z}}(t,T),g(X(t),\xi (t,T))\right) |{\mathscr {F}}_t\right] =H\left( t,{\mathscr {Y}}(t),X(t)\right) . \end{aligned}$$

As Assumption (LS) holds, we can apply Itô’s formula and obtain

$$\begin{aligned} dV^*(t)=&\,dH\left( t,{\mathscr {Y}}(t),X(t)\right) \\ =&\, H_t\left( t,{\mathscr {Y}}(t),X(t)\right) dt + H_y\left( t,{\mathscr {Y}}(t),X(t)\right) d{\mathscr {Y}}(t) +H_x\left( t,{\mathscr {Y}}(t),X(t)\right) dX(t)\\&+\left[ -H_{yx}\left( t,{\mathscr {Y}}(t),X(t)\right) {\mathscr {Y}}(t)\gamma (t)\sigma _X(t,X(t))+\frac{1}{2}{\mathscr {Y}}(t)^2\gamma (t)^2H_{yy}\left( t,{\mathscr {Y}}(t),X(t)\right) \right. \\&\left. +\frac{1}{2}\sigma _X(t,X(t))^2H_{xx}\left( t,{\mathscr {Y}}(t),X(t)\right) \right] dt\\ =&\,\left[ \vphantom {\left. +\frac{1}{2}\sigma _X(t,X(t))^2H_{xx}\left( t,{\mathscr {Y}}(t),X(t)\right) \right] }H_t\left( t,{\mathscr {Y}}(t),X(t)\right) -r(t){\mathscr {Y}}(t)H_y\left( t,{\mathscr {Y}}(t),X(t)\right) +\mu _X(t,X(t))H_x\left( t,{\mathscr {Y}}(t),X(t)\right) \right. \\&\left. -H_{yx}\left( t,{\mathscr {Y}}(t),X(t)\right) {\mathscr {Y}}(t)\gamma (t)\sigma _X(t,X(t))+\frac{1}{2}{\mathscr {Y}}(t)^2\gamma (t)^2H_{yy}\left( t,{\mathscr {Y}}(t),X(t)\right) \right. \\&\left. +\frac{1}{2}\sigma _X(t,X(t))^2H_{xx}\left( t,{\mathscr {Y}}(t),X(t)\right) \right] dt\\&+\left[ -H_y(t,{\mathscr {Y}}(t)){\mathscr {Y}}(t)\gamma (t) +H_x(t,{\mathscr {Y}}(t)) \sigma _X(t,X(t))\right] dW(t). \end{aligned}$$

Comparing the coefficients of the diffusion terms of this SDE and (2), we receice

$$\begin{aligned} V^*(t)\pi (t)\sigma (t)&=\,-H_y\left( t,{\mathscr {Y}}(t),X(t)\right) {\mathscr {Y}}(t)\gamma (t)+H_x\left( t,{\mathscr {Y}}(t),X(t)\right) \sigma _X(t,X(t)),\\ \end{aligned}$$

which completes the proof. $\square$

### Proof of Corollary 1

We have ${\hat{U}}_\omega (v)={\bar{U}}(v)$ since the liabilities are replicable with

$$\begin{aligned} {\bar{U}}(v) = U(v -\psi _L L(T,v)) = \frac{1}{1 - b}(v -\psi _L\left( vL_1(T)+ L_2(T)\right) )^{1 - b},\,v>{\hat{v}}_0(\omega )=\frac{\psi _LL_2(T)}{1-\psi _LL_1(T)} \end{aligned}$$

and

$$\begin{aligned} {\bar{U}}'(v)=(1-\psi _LL_1(T))(v-\psi _L(vL_1(T)+L_2(T)))^{-b},\,v>\frac{\psi _LL_2(T)}{1-\psi _LL_1(T)}. \end{aligned}$$

Since ${\bar{U}}'\left( \frac{\psi _LL_2(T)}{1-\psi _LL_1(T)}\right) =\infty$, the inverse of ${\bar{U}}'$ is given by

13 $$\begin{aligned} {\bar{I}}(y) = \frac{1}{1-\psi _LL_1(T)}\left( \left( \frac{y}{1-\psi _LL_1(T)}\right) ^{-\frac{1}{b}} + \psi _L L_2(T)\right) . \end{aligned}$$

Hence, given $v_0\ge {\mathbb {E}}\left[ {\tilde{Z}}(T)\frac{\psi _LL_2(T)}{1-\psi _LL_1(T)}\right]$, the optimal solution $V^*(T)$ from Theorem 1 is given by $V^*(T) = {\bar{I}}(Y(v_0){\tilde{Z}}(T))$, which reads as in the statement of the corollary. Furthermore,

$$\begin{aligned} H(0,y,X(0))={\mathbb {E}}\left[ \frac{{\tilde{Z}}(T)}{1-\psi _LL_1(T)}\left( \left( \frac{y{\tilde{Z}}(T)}{1-\psi _LL_1(T)}\right) ^{-\frac{1}{b}} + \psi _L L_2(T)\right) \right] \end{aligned}$$

and

$$\begin{aligned} H(0,Y(v_0),X(0))=v_0 \Leftrightarrow Y(v_0)=\left( \frac{v_0-{\mathbb {E}}\left[ {\tilde{Z}}(T)\frac{\psi _LL_2(T,X(T))}{1-\psi _LL_1(T,X(T))}\right] }{{\mathbb {E}}\left[ \left( \frac{{\tilde{Z}}(T)}{1-\psi _LL_1(T,X(T))}\right) ^{\frac{b-1}{b}}\right] }\right) ^{-b}. \end{aligned}$$

To obtain the optimal investment strategy, we apply Theorem 2 and calculate

$$\begin{aligned} V^*(t)=&\,H(t,{\mathscr {Y}}(t),X(t))={\mathbb {E}}\left[ {\tilde{Z}}(t,T){\bar{I}} ({\mathscr {Y}}(T))|{\mathscr {F}}_t\right] \\ =&\, {\mathbb {E}}\left[ \frac{{\tilde{Z}}(t,T)}{1-\psi _LL_1(T,X(T))}\left( \left( \frac{{\mathscr {Y}}(t){\tilde{Z}}(t,T)}{1-\psi _LL_1(T,X(T))}\right) ^{-\frac{1}{b}}+\psi _LL_2(T,X(T))\right) \bigg |{\mathscr {F}}_t\right] , \end{aligned}$$

which can be written as in the statement. Then,

14 $$\begin{aligned}&H_x(t,{\mathscr {Y}}(t),X(t))\nonumber \\&\quad ={\mathbb {E}}\left[ {\tilde{Z}}(t,T)\left( \frac{1}{b}-1\right) (-\psi _LL_{1,x}(T,X(T)))g_x(X(t),\xi (t,T)))\right. \nonumber \\&\qquad \cdot \left. \left[ 1-\psi _LL_1(T,g(X(t),\xi (t,T)))\right] ^{\frac{1}{b}-2}\left( {\mathscr {Y}}(t){\tilde{Z}}(t,T)\right) ^{-\frac{1}{b}}\bigg |{\mathscr {F}}_t\right] \nonumber \\&\qquad + {\mathbb {E}}\left[ {\tilde{Z}}(t,T)\frac{\psi _LL_{2,x}(T,X(T)g_x(X(t),\xi (t,T))}{1-\psi _LL_1(T,X(T))}\right. \nonumber \\&\qquad \left. +{\tilde{Z}}(t,T)\frac{\psi _LL_2(T,X(T))\psi _LL_{1,x}(T,X(T))g_x(X(t),\xi (t,T)))}{(1-\psi _LL_1(T,X(T)))^2}\bigg |{\mathscr {F}}_t\right] , \end{aligned}$$

where expectation and differentiation can be interchanged due to dominated convergence with Assumption (L2). Finally,

15 $$\begin{aligned} -H_y(t,{\mathscr {Y}}(t),X(t)){\mathscr {Y}}(t)\gamma (t)&={\mathbb {E}}\left[ {\tilde{Z}}(t,T)\frac{\partial }{\partial {\mathscr {Y}}(t)}{\hat{I}}_\omega ({\mathscr {Y}}(T))\big |{\mathscr {F}}_t\right] {\mathscr {Y}}(t)\gamma (t)\nonumber \\&= \frac{\gamma (t)}{b}{\mathscr {Y}}(t)^{-\frac{1}{b}}{\mathbb {E}}\left[ \left( \frac{{\tilde{Z}}(t,T)}{1-\psi _LL_1(T,g(X(t),\xi (t,T)))}\right) ^{\frac{b-1}{b}}\bigg |{\mathscr {F}}_t\right] \nonumber \\&=\frac{\gamma (t)}{b}\left( V^*(t)-\psi _L{\mathbb {E}}\left[ {\tilde{Z}}(t,T)\frac{L_2(T,X(T))}{1-\psi _LL_1(T,X(T))}\big |{\mathscr {F}}_t\right] \right) . \end{aligned}$$

Applying Theorem 2 and inserting (14) as well as (15), we have

$$\begin{aligned} \pi ^*(t)=&\,\frac{1}{\sigma (t)V^{*}(t)}\left[ -H_y\left( t,{\mathscr {Y}}(t),X(t)\right) {\mathscr {Y}}(t)\gamma (t)+H_x\left( t,{\mathscr {Y}}(t),X(t)\right) \sigma _X(t,X(t))\right] \\ =&\,\pi _M(t)-\frac{1}{\sigma (t)V^{*}(t)}\cdot \frac{\gamma (t)\psi _L}{b}{\mathbb {E}}\left[ {\tilde{Z}}(t,T)\frac{L_2(T,X(T))}{1-\psi _LL_1(T,X(T))}\big |{\mathscr {F}}_t\right] \\&+ \pi _{PL}(t)+\frac{\sigma _X(t,X(t))}{\sigma (t)V^{*}(t)}{\mathbb {E}}\left[ {\tilde{Z}}(t,T)\frac{\psi _LL_{2,x}(T,X(T)g_x(X(t),\xi (t,T))}{1-\psi _LL_1(T,X(T))}\bigg |{\mathscr {F}}_t\right] +\pi _{mi}(t)\end{aligned}$$

$$\begin{aligned}=&\,\pi _M(t)+\pi _{PL}(t)-\frac{1}{\sigma (t)V^*(t)}\cdot \frac{\gamma (t)\psi _L}{b}{\mathbb {E}}\left[ {\tilde{Z}}(t,T)\frac{L_2(T,X(T))}{1-\psi _LL_1(T,X(T))}\big |{\mathscr {F}}_t\right] \\&+ \frac{\psi _L}{\sigma (t)V^{*}(t)}{\mathbb {E}}\left[ {\tilde{Z}}(t,T)\frac{\sigma _X(t,X(t))L_{2,x}(T,X(T)g_x(X(t),\xi (t,T))}{1-\psi _LL_1(T,X(T))}\bigg |{\mathscr {F}}_t\right] +\pi _{mi}(t)\\ =&\, \pi _M(t)+\pi _{PL}(t)+\pi _{IL}(t)+\pi _{mi}(t), \end{aligned}$$

with $\pi _M(t),\,\pi _{PL}(t),\,\pi _{IL}(t)$ and $\pi _{mi}(t)$ as in the theorem statement. $\square$

### Proof of Corollary 2

We apply Theorem 2 and compute

$$\begin{aligned}&H\left( t,{\mathscr {Y}}(t),X(t)\right) \\&\quad ={\mathscr {Y}}(t)^{-\frac{1}{b}}{\mathbb {E}}\left[ {\tilde{Z}}(t,T)^{\frac{b-1}{b}}|{\mathscr {F}}_t\right] +\psi _L{\mathbb {E}}\left[ {\tilde{Z}}(t,T)L(T,g(X(t),\xi (t,T)))|{\mathscr {F}}_t\right] \\&\quad ={\mathscr {Y}}(t)^{-\frac{1}{b}}{\mathbb {E}}\left[ {\tilde{Z}}(t,T)^{\frac{b-1}{b}}|{\mathscr {F}}_t\right] +\psi _L L(0){\mathbb {E}}\left[ {\tilde{Z}}(t,T)\min (X(T),K)|{\mathscr {F}}_t\right] . \end{aligned}$$

With

$$\begin{aligned}&K \ge X(T)= X(t) e^{ \left( {\hat{\mu }}_X - \frac{1}{2} {\hat{\sigma }}_X^2 \right) (T-t) + {\hat{\sigma }}_X \left( W(T) - W(t) \right) }\\&\quad \Leftrightarrow \frac{ W(T) - W(t)}{\sqrt{T-t}} \le \frac{\log \left( \frac{K}{X(t)}\right) - \left( {\hat{\mu }}_X -\frac{1}{2} {\hat{\sigma }}_X^2 \right) (T-t) }{{\hat{\sigma }}_X\sqrt{T-t}}=:-{\bar{d}}_2(t), \end{aligned}$$

$$\begin{aligned}&{\mathbb {E}}\left[ {\tilde{Z}}(t,T)\min (X(T),K)|{\mathscr {F}}_t\right] \\&\quad =e^{-r(T-t)}K-{\mathbb {E}}\left[ {\tilde{Z}}(t,T)\max \left( K-X(T),0\right) |{\mathscr {F}}_t\right] \\&\quad =e^{-r(T-t)}\left( K-\int _{-\infty }^{-{\bar{d}}_2(t)}e^{-\frac{\gamma ^2}{2}(T-t)-\gamma \sqrt{T-t}u}K\phi (u)du\right. \\&\qquad \left. +X(t)\int _{-\infty }^{-{\bar{d}}_2(t)}e^{-\frac{\gamma ^2}{2}(T-t)-\gamma \sqrt{T-t}u}e^{\left( {\hat{\mu }}_X-\frac{1}{2}{\hat{\sigma }}_X^2\right) (T-t)+{\hat{\sigma }}_X\sqrt{T-t}u}\phi (u)du\right) \\&\quad =e^{-r(T-t)}\left( K-\int _{-\infty }^{-{\bar{d}}_2(t)}K\phi (u+\gamma \sqrt{T-t})du\right. \\&\qquad \left. +X(t)e^{({\hat{\mu }}_X-{\hat{\sigma }}_X\gamma )(T-t)}\int _{-\infty }^{-{\bar{d}}_2(t)}\phi \left( u+(\gamma -{\hat{\sigma }}_X)\sqrt{T-t}\right) du\right)\end{aligned}$$

$$\begin{aligned}&\quad =e^{-r(T-t)}\left( K-\int _{-\infty }^{-d_2(t)}K\phi (u)du+X(t)e^{\left( {\hat{\mu }}_X-{\hat{\sigma }}_X\gamma \right) (T-t)}\int _{-\infty }^{-d_2(t)}\phi \left( u-{\hat{\sigma }}_X\sqrt{T-t}\right) du\right) \\&\quad =e^{-r(T-t)}\left( K-K{\varPhi }(-d_2(t))+X(t)e^{\left( {\hat{\mu }}_X-{\hat{\sigma }}_X\gamma \right) (T-t)}{\varPhi }(-d_1(t))\right) \\&\quad =e^{-r(T-t)}\left( K{\varPhi }(d_2(t))+X(t)e^{\left( {\hat{\mu }}_X-{\hat{\sigma }}_X\gamma \right) (T-t)}{\varPhi }(-d_1(t))\right) , \end{aligned}$$

where $d_1(t)$ and $d_2(t)$ are as stated in the theorem and $\phi (x)$ denoting the density function of a standard normally distributed random variable. Using

$$\begin{aligned} \frac{\partial }{\partial X(t)}d_1(t)=\frac{\partial }{\partial X(t)}d_2(t)=\frac{1}{X(t){\hat{\sigma }}_X\sqrt{T-t}}, \end{aligned}$$

we receive

$$\begin{aligned}&H_x\left( t,{\mathscr {Y}}(t),X(t)\right) \\&\quad =\frac{\partial }{\partial X(t)}\psi _L L(0)e^{-r(T-t)}\left( K{\varPhi }(d_2(t))+X(t)e^{\left( {\hat{\mu }}_X-{\hat{\sigma }}_X\gamma \right) (T-t)}{\varPhi }(-d_1(t))\right) \\&\quad = \psi _LL(0)e^{-r(T-t)}\left( \frac{K\phi (d_2(t))}{X(t){\hat{\sigma }}_X\sqrt{T-t}}+e^{\left( {\hat{\mu }}_X-{\hat{\sigma }}_X\gamma \right) (T-t)}{\varPhi }(-d_1(t))-\frac{e^{\left( {\hat{\mu }}_X-{\hat{\sigma }}_X\gamma \right) (T-t)}\phi (-d_1(t))}{{\hat{\sigma }}_X\sqrt{T-t}}\right) \\&\quad = \psi _LL(0)e^{-r(T-t)}e^{\left( {\hat{\mu }}_X-{\hat{\sigma }}_X\gamma \right) (T-t)}{\varPhi }(-d_1(t)), \end{aligned}$$

since $$\phi (x)=\phi (-x)$$ and

$$\begin{aligned} \phi (d_2(t))=&\,\frac{1}{\sqrt{2\pi }}e^{-\frac{d_2(t)^2}{2}}=\frac{1}{\sqrt{2\pi }}e^{-\frac{(d_1(t)-{\hat{\sigma }}_X\sqrt{T-t})^2}{2}}\\ =&\,\frac{1}{\sqrt{2\pi }}e^{-\frac{d_1(t)^2-2d_1(t){\hat{\sigma }}_X\sqrt{T-t}+{\hat{\sigma }}_X^2(T-t)}{2}}\\ =&\,\frac{1}{\sqrt{2\pi }}e^{-\frac{d_1(t)^2}{2}}e^{\log \left( \frac{X(t)}{K}\right) +\left( {\hat{\mu }}_X-{\hat{\sigma }}_X\gamma +\frac{1}{2}{\hat{\sigma }}_X^2\right) (T-t)-\frac{1}{2}{\hat{\sigma }}_X^2(T-t)}\\ =&\,\frac{X(t)}{K}\frac{1}{\sqrt{2\pi }}e^{-\frac{d_1(t)^2}{2}}e^{\left( {\hat{\mu }}_X-{\hat{\sigma }}_X\gamma \right) (T-t)}=\frac{X(t)}{K}\phi (d_1(t))e^{\left( {\hat{\mu }}_X-{\hat{\sigma }}_X\gamma \right) (T-t)}. \end{aligned}$$

Using (15), we receive

$$\begin{aligned}&-H_y(t,{\mathscr {Y}}(t),X(t)){\mathscr {Y}}(t)\gamma (t)=\frac{\gamma (t)}{b}\left( V^*(t)-\psi _L{\mathbb {E}}\left[ {\tilde{Z}}(t,T)\min (X(T),K)\big |{\mathscr {F}}_t\right] \right) . \end{aligned}$$

The statement then follows directly from Theorem 2. $\square$

### Proof of Theorem 3

For the power utility case with asset participation type liabilities, we have

$$\begin{aligned} {\hat{U}}_\omega (v)=&\,{\mathbb {E}}\left[ \tfrac{1}{1-b}\left( v-\psi _LvL_1(T)\right) ^{1-b}\big |{\mathscr {F}}_T\right] =\frac{v^{1-b}}{1-b}{\mathbb {E}}\left[ \left( 1-\psi _LL_1(T)\right) ^{1-b}\big |{\mathscr {F}}_T\right] \end{aligned}$$

and thus, we can directly calculate

$$\begin{aligned} {\hat{U}}_\omega '(v)=v^{-b}{\mathbb {E}}\left[ \left( 1-\psi _LL_1(T)\right) ^{1-b}\big |{\mathscr {F}}_T\right] . \end{aligned}$$

Using (L2.1) and $k_1,\psi _L\in (0,1)$, we get ${\hat{v}}_0(\omega )=0$ and thus ${\hat{U}}'_\omega ({\hat{v}}_0(\omega ))={\hat{U}}'_\omega (0)=0$. Hence,

$$\begin{aligned} {\hat{I}}_\omega (y)=\left( {\varDelta }_\omega y\right) ^{-\frac{1}{b}}, \end{aligned}$$

for all $v>0$ with

$$\begin{aligned} {\varDelta }_\omega =\left( {\mathbb {E}}\left[ \left( 1-\psi _LL_1(T)\right) ^{1-b}|{\mathscr {F}}_T\right] \right) ^{-1} \end{aligned}$$

and

$$\begin{aligned} H(0,y,X(0))={\mathbb {E}}\left[ {\tilde{Z}}(T){\hat{I}}_\omega (y{\tilde{Z}}(T))\right] ={\mathbb {E}}\left[ {\tilde{Z}}(T)\left( {\varDelta }_\omega y{\tilde{Z}}(T)\right) ^{-\frac{1}{b}}\right] . \end{aligned}$$

Thus, $Y(v_0)$ is given by

$$\begin{aligned} H(0,Y(v_0),X(0))&={\mathbb {E}}\left[ {\tilde{Z}}(T)V^*(T)\right] =\, {\mathbb {E}}\left[ {\tilde{Z}}(T)\left( {\varDelta }_\omega Y(v_0){\tilde{Z}}(T) \right) ^{-\frac{1}{b}}\right] =v_0\\&\Leftrightarrow v_0= \,Y(v_0)^{-\frac{1}{b}}{\mathbb {E}}\left[ {\tilde{Z}}(T)\left( {\varDelta }_\omega {\tilde{Z}}(T) \right) ^{-\frac{1}{b}}\right] \\&\Leftrightarrow Y(v_0)=\,\left( \frac{1}{v_0}{\mathbb {E}}\left[ {\tilde{Z}}(T)\left( {\varDelta }_\omega {\tilde{Z}}(T) \right) ^{-\frac{1}{b}}\right] \right) ^{b}. \end{aligned}$$

Finally,

$$\begin{aligned} V^*(T)={\hat{I}}(Y(v_0){\tilde{Z}}(T)). \end{aligned}$$

$$\square $$

### Proof of Theorem 4

We apply Theorem 2 to the optimal wealth from Theorem 3. We have

$$\begin{aligned} V^*(t)=H(t,{\mathscr {Y}}(t),X(t))=&\,{\mathbb {E}}\left[ {\tilde{Z}}(t,T){\hat{I}}_\omega ({\mathscr {Y}}(T))|{\mathscr {F}}_t\right] \\ =&\, {\mathbb {E}}\left[ {\tilde{Z}}(t,T)\left( {\varDelta }_\omega (X(T)){\mathscr {Y}}(T)\right) ^{-\frac{1}{b}}|{\mathscr {F}}_t\right] \\ =&\, {\mathbb {E}}\left[ {\tilde{Z}}(t,T)\left( {\varDelta }_\omega \left( g(X(t),\xi (t,T))\right) {\mathscr {Y}}(t){\tilde{Z}}(t,T)\right) ^{-\frac{1}{b}}|{\mathscr {F}}_t\right] . \end{aligned}$$

For the calculation of $H_x$ and $H_y$, the expectation and differentiation can be interchanged by dominated convergence due to Assumption (L2). Hence,

$$\begin{aligned}&-H_y(t,{\mathscr {Y}}(t),X(t)){\mathscr {Y}}(t)\gamma (t)\\&\quad =-{\mathscr {Y}}(t)\gamma (t)\frac{\partial }{\partial {\mathscr {Y}}(t)}{\mathbb {E}}\left[ {\tilde{Z}}(t,T)\left( {\varDelta }_\omega \left( g(X(t),\xi (t,T))\right) {\mathscr {Y}}(t){\tilde{Z}}(t,T)\right) ^{-\frac{1}{b}}|{\mathscr {F}}_t\right] \\&\quad =\frac{\gamma (t)}{b}{\mathbb {E}}\left[ {\tilde{Z}}(t,T)\left( {\varDelta }_\omega \left( g(X(t),\xi (t,T))\right) {\mathscr {Y}}(t){\tilde{Z}}(t,T)\right) ^{-\frac{1}{b}}|{\mathscr {F}}_t\right] \\&\quad =\frac{\gamma (t)}{b}V^*(t) \end{aligned}$$

and

$$\begin{aligned} H_x(t,{\mathscr {Y}}(t),X(t))=&\,\frac{\partial }{\partial X(t)}{\mathbb {E}}\left[ {\tilde{Z}}(t,T)\left( {\varDelta }_\omega \left( g(X(t),\xi (t,T))\right) {\mathscr {Y}}(t){\tilde{Z}}(t,T)\right) ^{-\frac{1}{b}}|{\mathscr {F}}_t\right] \\ =&\,{\mathscr {Y}}(t)^{-\frac{1}{b}}{\mathbb {E}}\left[ {\tilde{Z}}(t,T)^{\frac{b-1}{b}}\frac{\partial }{\partial X(t)}\left( {\varDelta }_\omega (g(X(t),\xi (t,T)))\right) ^{-\frac{1}{b}}|{\mathscr {F}}_t\right] \end{aligned}$$

The statement follows with Theorem 2. $\square$

### Proof of Corollary 3

For liabilities of the form (PLUCB), the corresponding random utility function is given by

$$\begin{aligned} {\hat{U}}_{\omega }(v) ={\mathbb {E}}\left[ U\left( v - \psi _L vL(0) \left( \beta _1 f(X(T)) + \beta _2 {\mathscr {U}}_1\right) \right) \big \vert {\mathscr {F}}_T\right] . \end{aligned}$$

For $K < \frac{1}{\beta _1} \left( \frac{1}{\psi _L L(0)} - \beta _2 c \right)$, we obtain with $f(X(T))\le K$ that

$$\begin{aligned} L_1(T,X(T),{\mathscr {U}}_1))= \psi _L L(0) \left( \beta _1 f(X(T)) + \beta _2 {\mathscr {U}}_1\right)<\psi _LL(0)(\beta _1K+\beta _2c)=:k_1<1. \end{aligned}$$

Hence, (L2.1) holds and ${\hat{U}}$ is well-defined for all $v>0$, i.e. ${\hat{v}}_0(\omega )\equiv 0$. The optimal terminal wealth is given by Theorem 4.1 with ${\varDelta }_\omega =\left( {\mathbb {E}}[\left( 1-\psi _LL_1(T)\right) ^{1-b}|{\mathscr {F}}_T]\right) ^{-1}$. First, we consider the case $b\ne 2$. Since *X*(*T*) is ${\mathscr {F}}_T$-measurable and ${\mathscr {U}}_1$ is independent of ${\mathscr {F}}_T$, it follows that

$$\begin{aligned} {\mathbb {E}}\left[ \left( 1-\psi _LL_1(T)\right) ^{1-b}|{\mathscr {F}}_T\right] = \frac{1}{c-a} \int _a^c \left( 1 - \psi _L L(0)\left( \beta _1 f(X(T)) + \beta _2 u \right) \right) ^{1-b} du, \end{aligned}$$

and consequently

$$\begin{aligned} \frac{1}{{\varDelta }_\omega }&= \frac{-1}{(c-a)(\psi _L L(0)\beta _2 )} \int _{ 1- \psi _L L(0) \left( \beta _1 f(X(T)) + \beta _2 a \right) }^{1 - \psi _L L(0)\left( \beta _1 f(X(T)) + \beta _2 c \right) } u^{1-b} du \\&= \tfrac{\left( 1- \psi _L L(0)\left( \beta _1 f(X(T)) + \beta _2 a \right) \right) ^{2-b} - \left( 1- \psi _L L(0)\left( \beta _1 f(X(T)) + \beta _2 c \right) \right) ^{2-b}}{(c-a) (\psi _L L(0)\beta _2 ) (2-b)}\,. \end{aligned}$$

Furthermore,

$$\begin{aligned} \frac{\partial }{\partial X(t)}{\varDelta }_\omega ^{-\frac{1}{b}}&=\frac{\partial }{\partial X(t)}\left( \tfrac{\left( 1- \psi _L L(0)\left( \beta _1 f(g(X(t),\xi (t,T))) + \beta _2 a \right) \right) ^{2-b} - \left( 1- \psi _L L(0)\left( \beta _1 f(g(X(t),\xi (t,T))) + \beta _2 c \right) \right) ^{2-b}}{(c-a) (\psi _L L(0)\beta _2 ) (2-b)}\right) ^{\frac{1}{b}}\\&=\frac{1}{b}\left( \frac{-\psi _LL(0)\beta _1f'(X(T))g_x(X(t),\xi (t,T))}{(c-a) \left( \psi _L L(0)\beta _2 \right) (2-b)}\right) \left[ \left( 1-\psi _LL(0)\left( \beta _1f(X(T))+\beta _2a\right) \right) ^{1-b}\right. \\&\quad \left. -\left( 1-\psi _LL(0)\left( \beta _1f(X(T))+\beta _2c\right) \right) ^{1-b} \right] \\&\quad \cdot (2-b) \left( \tfrac{\left( 1- \psi _L L(0)\left( \beta _1 f(X(T)) + \beta _2 a \right) \right) ^{2-b} - \left( 1- \psi _L L(0)\left( \beta _1 f(X(T)) + \beta _2 c \right) \right) ^{2-b}}{(c-a) (\psi _L L(0)\beta _2 ) (2-b)}\right) ^{\frac{1}{b}-1}, \end{aligned}$$

which can be written as in the statement. In case $b=2$, the proof works analogously, but we have

$$\begin{aligned} \frac{1}{{\varDelta }_\omega }=\frac{1}{(c-a)(\psi _L L(0)\beta _2)}\log \left( \frac{1 - \psi _L L(0)\left( \beta _1 f(X(T)) + \beta _2 a \right) }{ 1- \psi _L L(0) \left( \beta _1 f(X(T)) + \beta _2 c \right) }\right) , \end{aligned}$$

respectively

$$\begin{aligned} {\varDelta }_\omega ^{-\frac{1}{b}}=\left( \frac{1}{(c-a)(\psi _L L(0)\beta _2 )}\log \left( \frac{1 - \psi _L L(0)\left( \beta _1 f(X(T)) + \beta _2 a \right) }{ 1- \psi _L L(0) \left( \beta _1 f(X(T)) + \beta _2 c \right) }\right) \right) ^{\frac{1}{b}} \end{aligned}$$

and

$$\begin{aligned} \frac{\partial }{\partial X(t)}{\varDelta }_\omega ^{-\frac{1}{b}}&=\frac{-\psi _LL(0)\beta _1f'(X(T))g_x(X(t),\xi (t,T))}{b\left( (c-a)(\psi _L L(0)\beta _2 )\right) ^{\frac{1}{b}}}\left( \log \left( \frac{1 - \psi _L L(0)\left( \beta _1 f(X(T)) + \beta _2 a \right) }{ 1- \psi _L L(0) \left( \beta _1 f(X(T)) + \beta _2 c \right) }\right) \right) ^{\frac{1}{b}-1}\\&\quad \cdot \left( \frac{1}{1 - \psi _L L(0)\left( \beta _1 f(X(T)) + \beta _2 a \right) }-\frac{1}{1 - \psi _L L(0)\left( \beta _1 f(X(T)) + \beta _2 c \right) }\right) . \end{aligned}$$

$$\square $$

### Proof of Corollary 4

The proof works along the proof of Corollary 3 with

$$\begin{aligned} {\varDelta }_\omega =\left( {\mathbb {E}}[\left( 1-\psi _LL_1(T)\right) ^{1-b}|{\mathscr {F}}_T]\right) ^{-1}=\left( 1-\psi _LL_1(T)\right) ^{b-1} \end{aligned}$$

since the liabilities are ${\mathscr {F}}_T$-measurable here. In this case, we obtain

$$\begin{aligned} \frac{\partial }{\partial X(t)}{\varDelta }_\omega ^{-\frac{1}{b}}&=\frac{\partial }{\partial X(t)}\left( \left( 1-\psi _LL(0)\beta _1 f(X(T))\right) ^{b-1}\right) ^{-\frac{1}{b}}\\&=\frac{\partial }{\partial X(t)}\left( 1-\psi _LL(0)\beta _1 f(g(X(t),\xi (t,T)))\right) ^{\frac{1}{b}-1}\\&= \left( 1-\psi _LL(0)\beta _1 f(X(T))\right) ^{\frac{1}{b}-2} \left( \frac{1}{b}-1\right) \left( -\psi _LL(0)\beta _1f'(X(T))\right) g_x(X(t),\xi (t,T)). \end{aligned}$$

$$\square $$

### Proof of Corollary 5

We proceed in a similar way as in the proof of Corollary 3. The random utility function for liabilities of the form (PLUCB*) is given by

$$\begin{aligned} {\hat{U}}_{\omega }(v) =&\, {\mathbb {E}}\left[ U(v - \psi _L v L(0) f(X(T){\mathscr {U}}_1(T))) \big \vert {\mathscr {F}}_T\right] . \end{aligned}$$

For $K < \frac{1}{\psi _L L(0)}$, we have with $f(X(T){\mathscr {U}}_1(T))\le K$

$$\begin{aligned} L_1(T,X(T),{\mathscr {U}}_1))= \psi _L L(0) f(X(T){\mathscr {U}}_1)\le \psi _LL(0)K=:k_1<1. \end{aligned}$$

Hence, (L2.1) holds and ${\hat{U}}$ is well-defined for all $v>0$, i.e. ${\hat{v}}_0(\omega )\equiv 0$. Again, we apply Theorem 3 and Theorem 4. It follows that

$$\begin{aligned} \frac{1}{{\varDelta }_\omega }=&\, \int _{-\infty }^{\infty } \left( 1 - \psi _L L(0) f\left( X(T) e^{ - \frac{1}{2} {\hat{\sigma }}_I^2 T + {\hat{\sigma }}_I \sqrt{T} u } \right) \right) ^{1-b} \phi (u)du, \end{aligned}$$

where $\phi (u)$ denotes the density of a standard normally distributed random variable. The statement follows by a straight-forward derivation using that expectation and differentiation can be interchanged here by dominated convergence due to (L2.1) and the properties of *f* as introduced in (PLUCB*). $\square$
